# Characterization of *Androctonus mauritanicus* venom and *in vitro* screening of SARS-CoV-2 entry inhibitors candidates

**DOI:** 10.3389/fphar.2025.1678606

**Published:** 2025-11-03

**Authors:** Reda Chahir, Jacob Galan, Hicham Hboub, Ahmed Salim Lahlou, Salma Chakir, Hinde Aassila, Reda Ben Mrid, Najat Bouchmaa, Reto Stöcklin, Rachid El Fatimy, Naoual Oukkache

**Affiliations:** ^1^ Laboratory of Venoms and Toxins, Pasteur Institute of Morocco, Casablanca, Morocco; ^2^ Faculty of Science and Technology, Agri-Food and Health Laboratory, Hassan First University of Settat, Settat, Morocco; ^3^ Department of Human Genetics, The University of Texas Rio Grande Valley School of Medicine, Brownsville, TX, United States; ^4^ Faculty of Medical Sciences, UM6P Hospitals, Mohammed VI Polytechnic University, Benguerir, Morocco; ^5^ Atheris Laboratories, Geneva, Switzerland

**Keywords:** *Androctonus mauritanicus*, scorpion venom, proteomics, mass spectrometry, antiviral peptides, SARS-CoV-2

## Abstract

Animal venom, known for its complex biochemical composition, presents a valuable source of therapeutic molecules, particularly for antiviral applications. Despite this potential, the industrial use of venom remains limited, with fewer than a dozen venom-derived compounds reaching commercial markets. This study underscores the significance of exploring venom’s natural diversity as a reservoir for novel bioactive compounds that could drive innovative drug development. We investigated the venom of the Moroccan black scorpion *Androctonus mauritanicus (Am)*, applying solid-phase extraction (SPE) and high-performance reversed-phase liquid chromatography (RP-HPLC) to fractionate the venom into 80 distinct samples. These fractions were subjected to detailed analysis using advanced mass spectrometry techniques, including ESI-MS, Q-TOF LC/MS, and Q-Exactive LC/MS. In total, 507 unique molecular masses were identified, with several fractions enriched in neurotoxins targeting ion channels (NaScTxs, KScTxs, CaScTxs, and ClScTxs), highlighting their therapeutic relevance. Fractions containing inhibitory molecules targeting the receptor-binding domain (RBD) of the SARS-CoV-2 Spike S protein were identified through *in vitro* validation via competitive ELISA, showing multiple levels of inhibitory potential. These findings demonstrate the antiviral activity of venom-derived molecules and reveal promising opportunities for venom-based industrial applications targeting SARS-CoV-2. In conclusion, this study not only emphasises the antiviral properties of specific venom molecules but also opens pathways for industrial drug development, offering potential tools to combat emerging viral diseases.

## 1 Introduction

Scorpion venoms are rich sources of bioactive peptides with demonstrated potential in treating various diseases, including cancer, microbial infections, and autoimmune disorders (Ortiz et al., 2015; Liu et al., 2018). While these venoms pose substantial public health risks in many regions, they also present exciting therapeutic opportunities; venoms from the Buthidae family, particularly *Androctonus* species, contain neurotoxins that modulate ion channels (Na^+^/K^+^/Ca^2+^), making them valuable for pain management and neurological research specifically amongst its other therapeutic potential (Hilal et al., 2023a). Table 1 lists some known therapeutic discoveries from different *Androctonus* subspecies:

**TABLE 1:** Selected therapeutic discoveries associated with various Androctonus subspecies.

Peptide	Subspecies	Target	Effect	References
AcrAP-1 & AcrAP-2 (NDBPs)	*Androctonus crassicauda*	Human neuroblastoma (SH-SY5Y)	Proliferation blocking	Zargan et al. (2011)
HC-AcrAP (cationic analogs)	*A. crassicauda*	Human breast cancer (MCF-7)		Zargan et al. (2011)
AaCTX	*Androctonus australis*	Human glioma cells U87	Cell migration and invasion inhibition	Rjeibi et al. (2011), Cheng et al. (2014)
Crude venom	*Androctonus.bicolor*	Human breast cancer (MDA-MB-231)	Cell motility and colony mitosis prevention	Al-Asmari et al. (2016)
Androctonin	*A. australis*	*Aspergillus brassicola, Stemphylium, Fusiarum culmorum, Botritis cinérea*	Antifungal activity	Ehret-Sabatier et al. (1996)
G-TI	*A. australis*	*Bacillus cereus* (Gram+)	Antibacterial activity	Zerouti et al. (2019)
Gonearrestide (P13)	*Androctonus mauritanicus*	Colorectal (HCT116) & glioma (U251) cells	Anti-proliferative; cell cycle arrest	Li et al. (2019)
Amm VIII	*A. mauritanicus*	Nav1.2 channel	Ion channel modulation	Abbas et al. (2013)
AaHIV	*A. australis*	DU145 prostate cancer (via Nav1.6 channel)	Anti-proliferative activity	BenAissa et al. (2020)
Mauriporin	*A. mauritanicus*	Prostate cancer cell lines	Anti-proliferative activity	Almaaytah et al. (2013)
AamAP1 AamAP2	*Androctonus amoreuxi*	Gram+ and Gram– bacteria	Antibacterial activity	Almaaytah et al. (2012)
AaeAP1 AaeAP2	*Androctonus aeneas*	*Candida albicans*	Antifungal activity	Du et al. (2015)

Among studied species, *Androctonus mauritanicus,* a scorpion endemic to North Africa, produces venom known for its highly potent neurotoxins (Hilal et al., 2023a). These bioactive components of their venom are increasingly recognized as valuable molecular tools for drug development. Indeed, venom-derived peptides have shown promising applications in pain modulation, antiviral therapies, and beyond, paving the way for novel therapeutic discoveries (Liu et al., 2018; Xia et al., 2024). Additionally, antimicrobial peptides (AMPs) from scorpion venom exhibit broad-spectrum activity against bacteria and fungi, with emerging evidence suggesting antiviral properties through mechanisms like viral membrane disruption (Xia et al., 2024). While *A. mauritanicus* venom has not yet been proven to have direct antiviral effects, its proteomic profile shares similarities with other scorpions such as *Androctonus australis* whose has been reported to exhibit antiviral effects against hepatitis C virus (HCV). In particular, crude venom from *A. australis* showed anti-HCV activity with a half-maximal inhibitory concentration (IC_50_) of 88.3 ± 5.8 μg/mL. This activity was preferentially directed against HCV and remained stable after heat treatment at 60 °C or metalloprotease inhibition, suggesting the involvement of heat-resistant venom peptides (El-Bitar et al., 2015). These studies strengthen the rationale for investigating *A. mauritanicus* peptides as potential inhibitors of SARS-CoV-2.

Proteomics plays a pivotal role in the identification and characterization of bioactive components within these complex venoms (Calvete, 2017). Advanced proteomic tools, such as mass spectrometry, enable detailed analysis of venom composition, uncovering a wide array of peptides and proteins. This approach, known as venomics, provides essential insights into the molecular diversity, structure, and biological function of venom molecules, facilitating the identification of candidates with therapeutic potential. Proteomics-driven venom research accelerates drug discovery by pinpointing molecules with targeted pharmacological activities (Oldrati et al., 2016).

In the field of antiviral therapies, venom peptides offer unique opportunities. Several studies have shown that peptides from animal venoms can inhibit viral replication or disrupt host-virus interactions (El Hidan et al., 2021), yet the antiviral potential of *A. mauritanicus* venom remains largely unexplored. Understanding how venom-derived molecules interact with viral proteins could unlock new therapeutic possibilities.

The COVID-19 pandemic, caused by SARS-CoV-2, has further emphasized the need for innovative antiviral treatments (Mahendran et al., 2024). While vaccination has been critical in controlling the spread of the virus, challenges such as production delays, unequal distribution, and the emergence of variants with partial immune escape have underscored the importance of developing complementary therapeutic strategies. Venom-derived peptides present a compelling option, as they can target key viral entry mechanisms, potentially blocking interactions between the SARS-CoV-2 spike protein (S protein) and host cell receptors (El Hidan et al., 2021). Furthermore, while current SARS-CoV-2 therapies mainly rely on small molecules or monoclonal antibodies (Iketani and Ho, 2024), their effectiveness can be compromised by viral mutations and resistance (Murdocca et al., 2024), There is therefore a clear unmet need for peptide-based inhibitors. Venom-derived peptides, with their stability, high specificity, and ability to interfere with protein–protein interactions, represent promising candidates to address this therapeutic gap (V et al., 2021). Thus, investigating animal venoms as sources of novel bioactive peptides offers a compelling strategy to develop innovative therapeutics, particularly in disorders where current treatments remain suboptimal (Kim et al., 2025).

This venomics study focuses on identifying peptides with potential antiviral activity by analyzing *A. mauritanicus* venom. Using mass spectrometry, we characterized the molecular composition of the venom and selected specific peptides for *in vitro* evaluation to identify their anti-viral capacity. Our findings demonstrate how proteomics provides a robust framework for the identification of venom-derived antiviral candidates. This work not only enhances the understanding of *A. mauritanicus* venom’s molecular diversity but also highlights its potential application in addressing viral threats like COVID-19. By exploring the therapeutic value of venom peptides, this research paves the way for alternative antiviral strategies and contributes to ongoing efforts in drug development.

## 2 Materials and methods

### 2.1 Venom extraction

**FIGURE 1:**
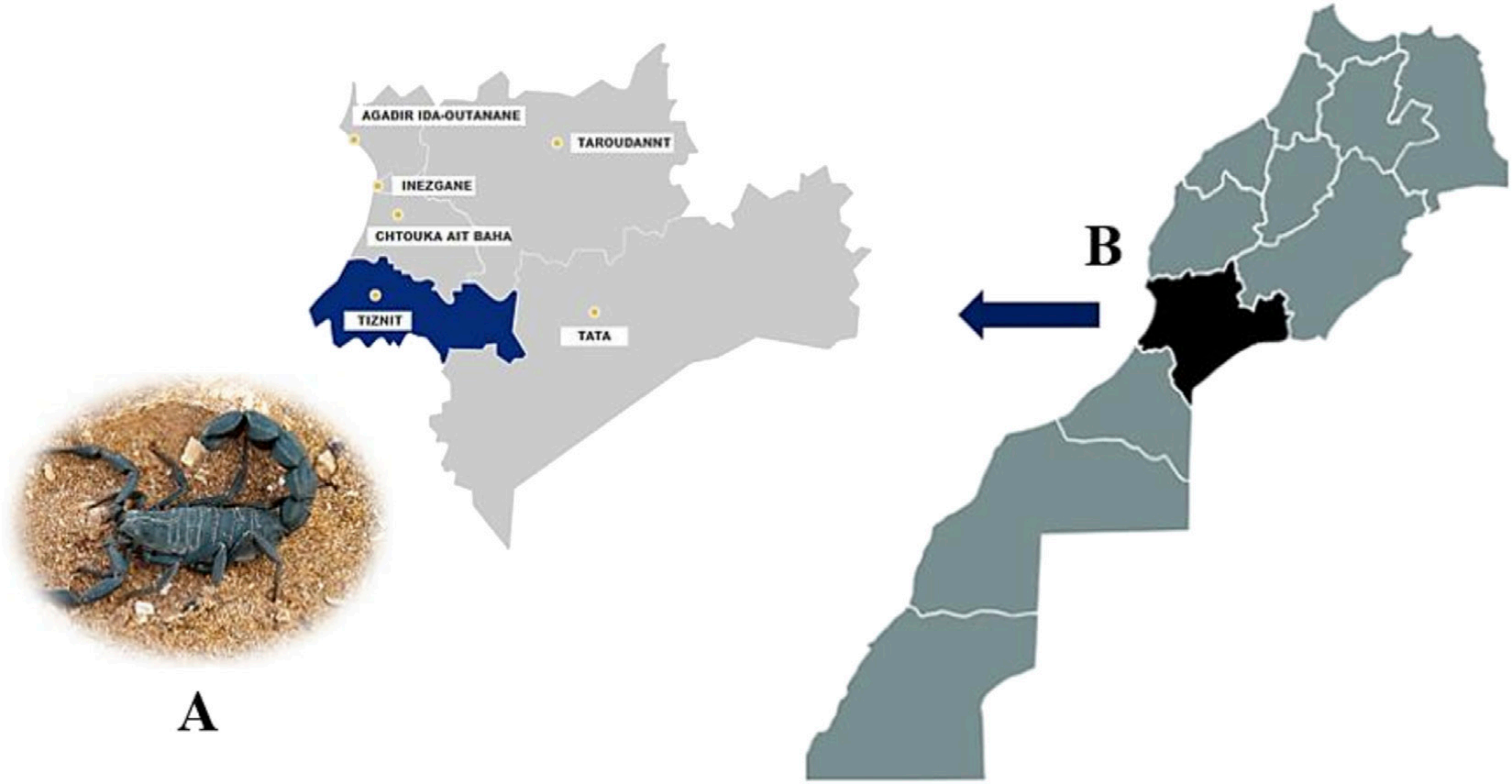
Geographical localization of the collected Am specimens **(A)**. The region of Tiznit in the western part of Morocco **(B)**, is known for its high-risk of scorpion envenomation.

*A. mauritanicus* scorpions were collected in Tiznit, Souss-Massa region (Figure 1), known for its high incidence of scorpion sting envenomation cases (El Oufir, 2019), and were preserved at the Pasteur Institute’s animal facility. Their venom was extracted by using electrical stimulation, and applying a low-voltage pulses of 12 V to the scorpions’ post-abdomen to facilitate venom ejection. Following venom collection, the pooled venom was centrifuged at 10,000 RPM for 10 min to separate impurities. The resulting supernatant was then lyophilized and stored at −80 °C, preserving its potency and bioactivity for future use (Yaqoob et al., 2016).

### 2.2 Venom preparation

#### 2.2.1 Venom solubilization

A quantity of 1 mg of *Am* venom was solubilized in 1 mL of solution A (0.1% trifluoroacetic acid (TFA)), and the mixture was centrifuged at a speed of 3,500 rpm for 5–10 min. Protein concentration was measured directly using a NanoDrop™ 2000 spectrophotometer (Thermo Fisher Scientific, Waltham, MA, United States of America) at an absorbance wavelength of 280 nm. An extinction coefficient of 10 (ε1%) was applied to estimate protein concentration. Venom samples were diluted appropriately, and each measurement was conducted in triplicate to ensure accuracy and reproducibility (Desjardins et al., 2009).

#### 2.2.2 Removal of salts and large molecules

The removal of salts and large molecules was achieved through solid-phase extraction (SPE), a technique used for the extraction, purification, and enrichment of venoms prior to analysis (Li et al., 2006). This method involves adsorbing the target compounds onto a stationary phase within a SEP-Pak cartridge, followed by their elution. Washing steps are employed to remove interfering substances. The SPE process comprises four main steps. First, the stationary phase was conditioned. Cartridges were mounted on 10 mL syringes and connected to a manifold, a vacuum chamber equipped with a peristaltic pump. The stationary phase was conditioned by adding 10 mL of methanol to clean and wet the phase, followed by 10 mL of solution A (0.1% TFA) to activate the functional groups on the surface. Next, the phase was loaded with 1 mg of venom, allowing compounds with a strong affinity for the stationary phase to be retained. This is followed by a washing step to eliminate molecules weakly retained by the stationary phase by adding 4 mL of solution A (0.1% TFA). Finally, the compounds of interest were eluted by percolating 3 mL of the elution solution (70% Solution B + 30% Solution A), breaking the interactions between the target compounds in the venom and the stationary phase. The yield of recovered proteins was calculated after estimating their concentration by measuring absorbance at 280 nm.

#### 2.2.3 Vacuum concentration using the SpeedVac

Following the solid-phase extraction process, the proteins obtained from the different venoms were initially frozen at −80 °C overnight to ensure their stability. Subsequently, they underwent concentration using the SpeedVac vacuum centrifugal concentrator. This step involved the removal of excess solvent under reduced pressure, allowing for the concentration of proteins. Once concentrated, the proteins were stored at −20 °C until further use.

### 2.3 Fractionation of the venom by reverse-phase high-performance liquid chromatography (RP-HPLC)

A total quantity of 30 mg of *A. mauritanicus* venom (1 mg for each run) was subjected to solid-phase extraction (SPE) and fractionated using the Alliance 2795 RP-HPLC system (Waters Corporation, Milford, MA, United States of America). Separation was achieved over 120 min on a Phenomenex C18 analytical column (250 mm, 4.6 mm, 5 µm) with a flow rate of 0.8 mL/min and a linear gradient of solvent B: from 2% to 70% over 113 min, followed by 90% for 6 min. Eluted proteins were detected at a wavelength of 280 nm, and the separated fractions were automatically collected into a 96-well plate. Fractions from each RP-HPLC run were pooled, dried, and stored at −80 °C until use.

### 2.4 Intact protein LC-MS

The identification of the average molecular masses of all purified fractions was performed on the triple quadrupole ESI-MS mass spectrometer (Micromass Quattro micro triple quadrupole). The fractions were dissolved in 100 µL of nebulization solvent (H_2_O/ACN/HCOOH, 49.8:50:0.2), and 10 µL of each was directly infused into the instrument using a Hamilton syringe. Ionization was conducted in positive mode in the ESI source, and the generated ions were separated in the Q-q-Q. MS scans with a mass-to-charge ratio (*m/z*) ranging from 500 to 1,500 Da were recorded. MassLynx 4.0 software (Waters-Micromass) was utilized for spectrum processing and molecular mass identification.

### 2.5 Identification and sequencing of peptides by mass spectrometry (nano-LC-MS/MS)

#### 2.5.1 Enzymatic digestion

##### 2.5.1.1 Reduction/alkylation

The fractions of interest were dissolved in 10 µL of ACN (30%) and reduced by 100 µL of the DTT solution (10 mM)/ammonium bicarbonate (50 mM, pH 8.3). The mixture was sonicated for 3 min, placed under a nitrogen atmosphere, and incubated at 60 °C for 2 h. Free sulfhydryl groups were blocked by iodoacetamide (IAA) (55 mM)/ammonium bicarbonate (50 mM, pH 8.3). Subsequently, the mixture was incubated for 20 min at room temperature and protected from light. This step concluded with the addition of 10 mM DTT to eliminate excess IAA and prevent overalkylation, followed by incubation for 1 hour at room temperature.

##### 2.5.1.2 Digestion

We used the enzymes trypsin and Lys-C individually for digestion: 1 µg of each enzyme, dissolved in 50 mM ammonium bicarbonate (pH 8.3), was added to each fraction. The samples underwent overnight incubation at 37 °C to allow for complete enzymatic digestion. To halt the enzymatic reaction, 10 µL of 5% formic acid was added to each sample. Subsequently, the entire mixture was evaporated using the SpeedVac concentrator to remove excess solvent and concentrate the peptides for further analysis.

#### 2.5.2 Quadrupole time-of-flight (Q-TOF) LC-MS/MS

The digested fractions were resuspended in 10 µL of 3% ACN/0.1% FA and then analyzed using the nano-LC1200 system coupled to the Q-TOF 6520 mass spectrometer (Agilent Technologies).

##### 2.5.2.1 Q-TOF data acquisition

The analysis was configured in data-dependent acquisition mode, where peptides were ionized in nano-ESI in positive mode with a voltage of 1850 V. Full autoMS1 scans with a mass-to-charge ratio (*m/z*) range from 200 to 1,700 and autoMS2 scans from 59 to 1,700 m*/z* were recorded. In each cycle, a maximum of 5 precursor ions sorted by their charge state (excluding singly charged precursor ions) were isolated and fragmented in the collision-induced dissociation (CID) cell. The collision cell energy was automatically adjusted based on the *m/z.*

##### 2.5.2.2 Q-TOF data processing

The generated data were processed using Peaks 7.5 software (Bioinformatics Solutions Inc., Waterloo, Canada). Peptides were identified by sequence homology using the UniProt database (https://www.uniprot.org) or by *de novo* sequencing for certain peptides. The search parameters were set as follows: precursor ion mass tolerance of 50 ppm and fragment ion mass tolerance of 0.3 Da. Enzyme specificity was set to trypsin for fractions digested with trypsin and Lys-C for those digested with Lys-C. For variable post-translational modifications, oxidation (M) (+15.9949 Da), carbamidomethylation (C) (+57.0214 Da), pyro-glu of Q and E, dehydration, and amidation were considered, while no fixed modifications were selected.

#### 2.5.3 Q-exactive LC-MS/MS

The analysis of the digested fractions was also subjected to analysis in the Orbitrap Q-Exactive Plus mass spectrometer (Thermo Fisher Scientific, Bremen) coupled with an UltiMate™ 3000 RSLC Nano HPLC system.

##### 2.5.3.1 Nano-HPLC

Fractionation was performed using the same parameters described in the previous paragraphs, except for an analytical column (PepMap RSLC C18, 75 μm × 25 cm, Thermo Scientific) and a flow rate of 300 nL/min.

##### 2.5.3.2 Q-exactive data acquisition

The analysis was configured in data-dependent acquisition mode, where peptides were ionized in positive mode with a spray voltage of 1.6 kV and a capillary temperature of 180 °C. MS spectra were acquired at a resolution of 60,000 with a mass-to-charge ratio (*m/z*) ranging from 300 to 1,500. The 5 most abundant precursor ions were selected for higher-energy collisional dissociation (HCD) fragmentation with a collision energy of 27. Singly charged ions and those with a charge state >7 were excluded, and MS/MS spectra were acquired at a resolution of 60,000 with a *m/z* range from 59 to 1,700.

##### 2.5.3.3 Q-exactive data processing

The processing of MS/MS data and peptide identification were performed following the same protocol described in the analysis by Q-TOF LC/MS.

### 2.6 The enzyme-linked immunosorbent assay (ELISA)

The purified fractions of the venom from the scorpion *A. mauritanicu*s, were assessed for their potential inhibitory effect on the binding between the receptor-binding domain (RBD) of the virus and hACE2. This evaluation was performed using the COVID-19 Spike-ACE2 Binding Assay Kit, generously provided by Atheris Laboratories (3 kits) (CoV-SACE2-1, RayBiotech Inc.), following the protocol outlined by the manufacturer. This type of assay is widely used to screen potential viral entry inhibitors. Previous studies have demonstrated that this type of approach can identify small molecules capable of effectively blocking the RBD–ACE2 interaction and thus preventing SARS-CoV-2 entry into cells (Zhang et al., 2022).

In the first step, the purified fractions were tested at two concentrations: 20 mg/mL and 40 mg/mL dry weight. These concentrations were selected based on several considerations. First, preliminary assessments of solubility ensured that these concentrations were compatible with the assay. Second, the low abundance of potential bioactive peptides in the complex venom fractions required relatively higher concentrations to detect inhibitory activity. Third, lower concentrations had been tested in preliminary experiments, but they did not produce significant inhibition of RBD–ACE2 binding. Finally, the observed dose-dependent inhibition at these concentrations indicates that the effects are specific and not due to nonspecific interactions. This approach is consistent with previous exploratory screenings of venom-derived peptides, which commonly use higher concentrations to identify fractions with potential activity (Chen et al., 2012). All concentrations were assessed in triplicates. Next, the analyzed fractions were mixed with recombinant hACE2 protein, while control samples received PBS instead of venom fractions. The mixture was then added to an ELISA plate pre-coated with SARS-CoV-2 RBD protein and incubated for 2 h at room temperature with shaking. After incubation, unbound ACE2 was removed by washing the plate.

For detection, binding was assessed using the reaction between an HRP-conjugated anti-ACE2 antibody and 3,3′,5,5′-tetramethylbenzidine (TMB). Absorbance was measured at 450 nm using a Mindray MW-12A microplate reader. Finally, Statistical analysis was performed using a one-way ANOVA followed by Dunnett’s *post hoc* test, comparing each fraction at each concentration with the negative control (PBS). Levels of significance are indicated as “ns” (not significant) (p < 0.01), and (p < 0.001).

### 2.7 *In Vivo* acute toxicity evaluation in mice

To assess the neurotoxic potential of the most bioactive venom fractions identified through ELISA-based inhibition of the ACE2–Spike protein interaction, intracerebroventricular (ICV) injections were performed in adult male Swiss mice (18–22 g). The selected fractions which demonstrated the highest inhibitory activity in ELISA assays were reconstituted in sterile phosphate-buffered saline (PBS) and injected in volumes not exceeding 10 µL per mouse.

Groups of 3–5 mice per fraction were used, with each group receiving escalating doses to evaluate the onset of clinical neurotoxic signs (e.g., tremors, ataxia, seizures, respiratory distress), and potential lethality. For lethal fractions, the median lethal dose (LD_50_) was calculated using the Reed and Muench method. Non-lethal doses and fractions were observed for sublethal neurotoxic signs and behavioral abnormalities during a 4-h acute phase and over 72 h post-injection. These data contribute to defining a safe dose window for future therapeutic development of the concerned fractions.

## 3 Results

### 3.1 Protein estimation

The results showed a protein yield of 0.98 mg/mL for *A. mauritanicus* venom, as estimated using the NanoDrop spectrophotometer.

### 3.2 Venom fractionation by reverse-phase High-Performance Liquid Chromatography (RP-HPLC)

**FIGURE 2:**
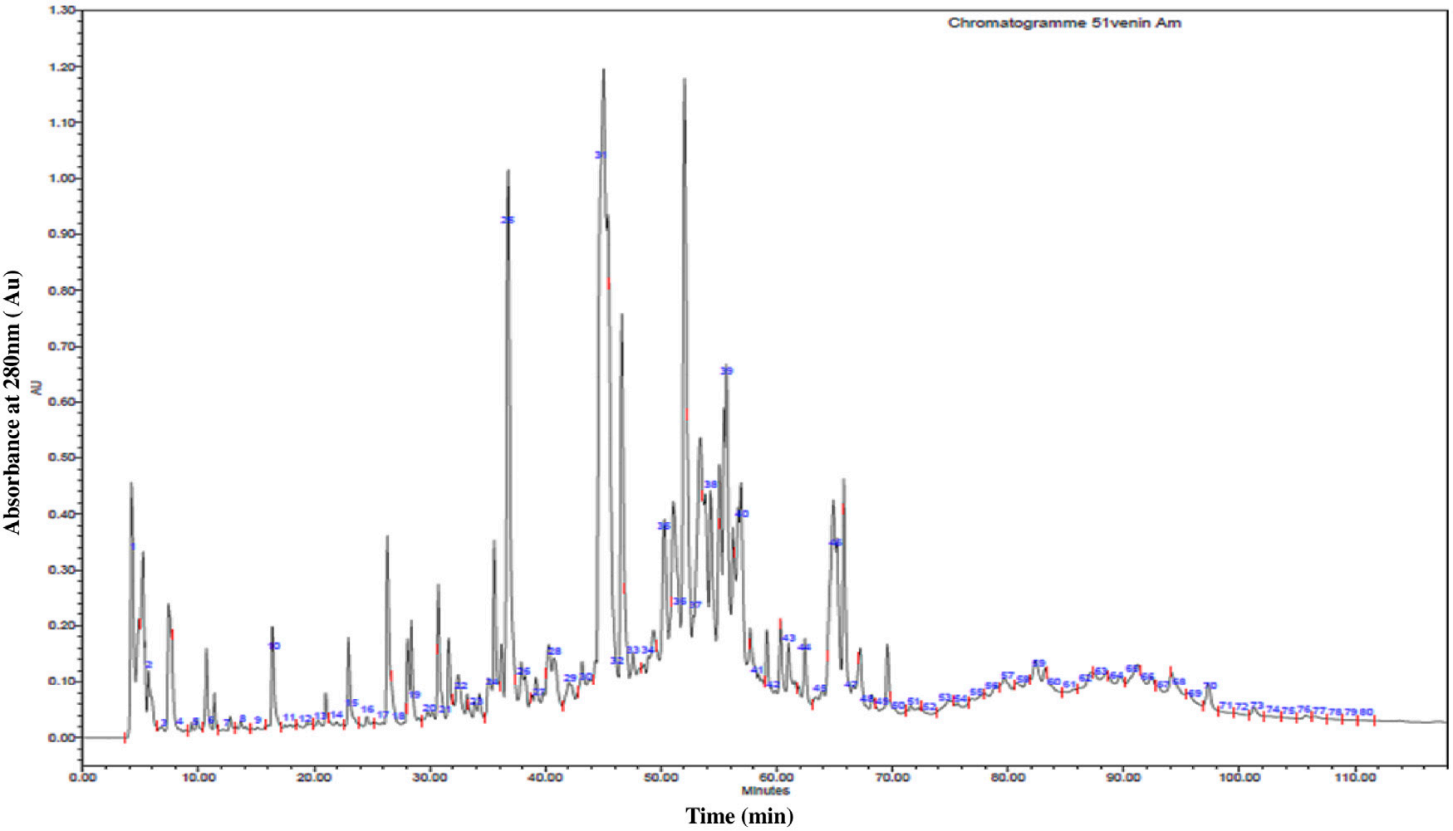
High-Performance Liquid Chromatography (RP-HPLC) profile of 1 mg of *A. mauritanicus* venom protein conducted with a linear gradient from solvent A (0.1% TFA in water) to 90% solvent B (0.1% TFA in acetonitrile) at a flow rate of 0.8 mL/min, and run for 120 min.

The chromatogram resulting from the fractionation of *A. mauritanicus* venom by RP-HPLC is illustrated in Figure 2. This profile represents a partial image of the various constituents of *A. mauritanicus* venom, comprising 80 different eluted fractions. This array of fractions provides insight into the richness and complexity of this venom. The majority of fractions were eluted with retention times ranging from 3.75 min to 70 min. The most intense fractions were eluted between 26 and 66 min, while the majority of minor peaks were observed within a time interval of 70–110 min. Among the most intense fractions, we find F25 (RT = 37 min), F31 (RT = 45.5 min), F32 (RT = 47 min), F37 (RT = 52 min), and F39 (RT = 55.5 min).

### 3.3 Identification of average molecular masses by mass spectrometry

The various fractions obtained through RP-HPLC underwent analysis using the triple quadrupole mass spectrometer “ESI-MS” to generate average molecular masses (Table 2). Data processing using MassLynx4 software identified a total of 507 molecular masses ranging from 200.18 Da to 10431 Da. Regarding the toxic profile, fractions of *A. mauritanicus* venom are rich in molecular masses ranging from 2001 to 5000 Da. These masses, corresponding to neurotoxins targeting potassium, chloride, or calcium channels, are the most abundant, comprising 51.29% of the total. Conversely, masses beyond 5001 Da, corresponding to neurotoxins targeting sodium channels, represent 22.86% of the total.

**TABLE 2:** List of the different average masses identified in the various fractions of *A. mauritanicus* venom.

Fractions	Masses
1	200.18, 228.43, 270.88, 306.94, 411.69, 443.55, 573.66, 709.69
2	228.36, 246.52, 280.75, 365.23, 573.66
3	212.25, 238.5, 254.66, 266.78, 290.93, 306.94, 421.46, 437.55, 1303.47
4	254.6, 418.73, 497.71, 536.93, 557.9, 574.92
5	207.19, 385.34
6	323.01, 385.36, 595.96
7	207.19, 385.37, 400.66
8	385.37, 400.66
9	207.19, 323.03, 385.37
10	290.86, 306.93, 323.0
11	306.94, 385.35
12	306.95, 368.5, 371.52, 385.37
13	207.18,222.36, 254.58, 254.58, 385.34,492.86
14	222.37, 306.95, 254.6,207.19,290.89,363.82,385.35,414.98
15	222.37, 254.59, 290.87,323.01,385.35,872.25, 254.57, 202
16	208.34, 222.36,254.58, 290.87, 323.01, 385.35, 744.34
17	208.38, 222.37, 254.58, 290.87, 323.01, 385.34
18	222.36, 254.58, 290.88, 323
19	3185, 3214, 3180.12, 3206.3, 2950.72, 2954, 6032, 10212, 10211, 3101.29
20	7559, 3214, 10212, 10211
21	3185, 3206.3, 8355
22	3185, 3214, 3180.12, 3206.3, 2954, 2874.46
23	2934.29, 2950.43, 2988.23, 3975.76, 3993.02
24	550.35, 2934.53, 2963.86, 2988.35, 3005.20, 3416.14, 3950.46, 4054.06, 4054.43, 4260.07
25	6145, 6175, 6512, 4121, 6161, 8218, 4376.27, 2950.72, 2935.29, 2874.46, 6032, 10212, 10211
26	514.35, 3197.9, 3850.96, 6913.93
27	1539.57, 1698.34, 2073.57, 3209.20, 4189.08, 2656.41
28	8218, 2950.72, 2942.58, 6032, 6307, 10431, 10212, 10192, 10211, 7177, 3607.3, 3673, 7176, 5398.1, 8355
29	3875, 3823.5, 6291, 6277, 3751.48, 4156, 4072, 4184.08, 3980.8, 6523, 3962, 4097, 4107, 4021, 6393, 6462, 4121.4, 4376.27, 10431, 10144, 10148, 7177, 7176, 5398.1, 8355
30	3627.65, 3665.52, 3682.19, 3719.31, 4206.79, 5224.74, 6591.18
31	8702, 8059, 9061, 8218, 4156, 4072, 4184.08, 3980.8, 6523, 3962, 4097, 4107, 4021, 6393, 6462, 4121.4, 4376.27, 10431, 10211, 3607.8, 3747.48, 3673, 5398.1
32	3607.88, 3627.42, 3665.40, 3681.31
33	8702, 9061, 8218, 10431, 10212, 10192, 10211, 3607.8, 3673, 1931.94, 1880.93, 5398.1
34	9571, 9496, 8702, 7301,7268, 9649,9366, 9426, 4130, 9412, 7212, 7334.93, 7373, 7226, 7465, 9511, 9524, 7253, 9174, 6872, 8059, 7301, 7733.59, 7468, 9061, 7539, 9283, 9398, 9334, 6918, 9211, 9320, 6882, 7178.27, 8218, 4156, 4072, 4184.08, 3980.8, 6523, 3962, 4097, 4107, 4021, 6393, 6462, 4121.4, 4376.27, 10431, 10144, 4005, 3607.8, 3673, 5398.1
35	9425, 9571, 9496,9068, 7343, 8702, 7301,7268,7194, 7211.3, 9649,9366, 9426, 4130, 8756, 9412, 7021, 9319, 9308, 7212, 7334.93, 7485, 7964, 7267, 9511, 6937, 9442, 9174, 7170.47, 6872, 8059,7040.5,7057, 7301, 7001.82, 7384, 7226, 9061, 6882, 9283, 9398, 7477, 7440, 9271, 7186, 9330, 9334, 6918, 9211, 9320, 6882, 10431, 10212, 10211, 7177, 4005, 3607.8, 3835, 3673, 7913, 7985, 7773
36	9571, 9496, 7268, 7194, 7211.3, 9649, 8756, 7021, 9319, 9308, 7240, 7267, 6872, 7040.5, 7001.82, 7382, 9061, 6882, 6845, 9312, 9330, 9296, 10431, 10212, 10211, 10144, 4005, 3607.8, 3673
37	6872, 7040.5, 7057, 6836.25, 6791.78, 6882, 10431, 10211, 10144, 4005, 3673
38	8702, 6872, 7040.5, 7001.82, 9061, 6882, 9351, 9019, 9312, 9330, 9936, 6918, 6652.15, 6592.5, 7641, 9296, 10431, 10144, 4005, 3607.8, 3673
39	7301, 6872, 7040.5, 9351, 9019, 9901, 9959, 9936, 6652.15, 7641, 7620.3, 10431, 4005, 3607.8, 3673, 7913, 7985, 7773
40	7020.57, 7293.31, 7383.47, 7662.26, 7677.99, 7797.82, 7948.83
41	7301, 6872, 7998, 7786, 8149.39, 9901, 9959, 9936, 7641, 10212, 10211, 4005, 3673
42	2542.55, 2762.53, 3172.12, 4419.48, 5194.61, 5577.46, 5857.4, 5884.81, 7045.13, 7061.68, 7420.22
43	3664.64, 3809.42, 7028.67
44	6872, 7040.5, 6882, 6845, 6836.25, 6791.78, 6918, 6892.4, 10431, 10212, 10211, 10144, 4005, 3673
45	6872, 7040.5, 6882, 6836.25, 6791.78, 10212, 10211, 4005, 3673, 7913, 7985, 7773
46	8702, 6872, 4005, 3673, 7913, 7985, 7773
47	3035.07, 6933.8
48	7785, 9574, 7721, 10212, 10211
49	3809.15, 3824.6, 3863.24, 7785
50	1525.12, 1851.69,2541.32,3050.45,3702.24, 5729.8, 6479.81
51	1348.87, 1419.01, 1547.58, 1842.14, 1982.35, 1998.64, 2135.36, 3538.45, 3910.64
52	1722.18, 2293.99, 2311.55, 2335.57, 2662.75, 3562.63, 3577.40, 3665.69, 4129.31 4698.28, 4996.71, 5900.00, 7160.87
53	4055.48, 4399.39, 4473.21, 5838.77, 5899.73, 6934.73, 7109.03
54	2839, 2968
55	1614.22, 1698.15, 2608.39, 3061.15
56	1542.38, 1849.48, 2013.42, 2041.26, 2049.06, 2571.94, 2738.67, 3524.11, 4099.11, 4310.19, 5769.12
57	1614.44, 1784.97, 2825.59, 3391.29, 3684.08, 4068.98
58	1889.19, 2003.54, 2066.50, 2109.32, 2442.24, 2682.6, 3226.6, 3356.51, 3608.46, 3642.97, 4600.16, 4431.93
59	2325.28, 2785.35, 2921.24, 3259.93, 4068.92
60	1956.09, 3505.12, 8682.28
61	2080.52, 2152.48
62	1753.45, 2200.34, 2631.63, 2932.89, 3760.41, 5118.16, 5132.78, 5263.27, 5670.88, 6140.99, 6396.99, 6700.58, 8955.78
63	574.07, 1076.2, 1303.91, 1305.99, 1336.37, 1545.78, 3482.77, 2831.11, 2921.24, 3579.87, 5725.43, 5303.84, 7000.04, 7281.16, 8274.75
64	1902.47, 2152.26, 2814.62, 2984.1, 3341.34,3482.27, 3579.86, 3912.45, 5706.40,
65	1193.67, 1614.1, 1721.96, 2336.51, 2492.56, 2717.42, 2885.76, 3260.84, 4829.64, 5268.65, 6673.50, 7508.48
66	1614.16, 1908.86, 1956.53, 2511.51, 2575.95, 2591.28, 2920.83, 2982.97, 3260.44, 3580.64, 5370.36
67	1722.43, 1956.40, 4707.66, 6414.55
68	1698.4, 1794.63, 2336.4, 2449.39, 2474.35, 3371.23, 3765.74, 7756.25, 8407.82
69	2152.01, 2336.05, 3913.6, 3963.25
70	1794.32, 2831.11, 2336.72, 4564.66,
71	1974.26, 2086.2, 2258.68, 2364.46, 2482.35, 2961.39, 3152.1, 3310.08, 3377.73, 3556.74, 3948.52, 4172.39, 4742.32, 5066.1, 5215.49, 5792.35, 6258.59, 6909.91, 7113.48, 8299.06
72	1321.12, 1757.45, 2429.18, 2636.17, 2636.17, 2735.7, 3185.77, 3302.81, 3514.89, 3647.6, 4247.69, 4559.5, 5272.34, 5309.61, 5471.4, 5668.08, 6151.06, 6371.53, 6383.2, 6383.3, 7433.46
73	1675.16, 2093.88, 3062.85, 4187.75, 5324.69, 7328.57
74	2144.37, 2807.84, 3216.55, 3855.34, 4679.74, 4819.18, 5360.92, 5615.69, 5783.02, 6433.11, 6551.63, 6746.85, 7505.29
75	3424.7, 4156.27, 5707.84, 6849.41, 7990.98, 8312.55
76	1788.4, 2683.2, 3667.49, 4471.99, 6261.01
77	1413.66, 1589.97, 2264.38, 2866.38, 2993.07, 3242.19, 3662.96, 3817.42
78	1833.96, 1963.61, 2125.02, 2184.86, 2336.66, 2361.12, 2415.66, 2443.97, 2537.03, 2691.23, 2715.33, 3102.8, 3217.04, 3591.15, 5910.26
79	1413.82, 1614.03, 2301.93, 2654.35, 2810.97, 2830.99, 2870.42, 2891.17, 3542.81, 3662.84, 4008.24, 4563.97, 5062.41, 5881.75, 8695.26
80	2952.67, 4173.81, 6261.99, 7305.65

Interestingly, 86 peptides corresponding to NaScTxs were characterized, 64 of which were identified as NaSctx alpha subfamily (α-NaScTxs) with a sequence coverage ranging from 9% for the peptide similar to Lipolysis-activating peptide 1-alpha chain (D9U2A4) to 86% for the peptides who present homology with the alpha-toxin Lqq4 (P01489), whereas, 23 peptides corresponding to as NaSctx beta subfamily (β-NaScTxs) with a sequence coverage from to 8% (Beta-insect depressant toxin BmKIT4; Q17230) to 39% (Insect toxin AaHIT5; P81504) (Table 3).

**TABLE 3:** The different NaScTxs identified in the fractions of *A. mauritanicus* venom.

Accession number	Name	Sequence	Coverage (%)	Fractions	MW
NaScTxs					
α-NaScTxs					
Q8I0K7	Depressant scorpion toxin BmKIM OS = *Mesobuthus martensii* GN = KIM2 PE = 2 SV = 1	MKLFLLLVFFASMLIDGLVNADGYIRGSNGCKISCLWGNEGCNKECKGFGAYYGYCWTWGLACWCEGLPDDKTWKSESNTCGGKK	6	F35	9,425
P17728	Alpha-insect toxin LqhaIT OS = *Leiurus quinquestriatus hebraeus* PE = 1 SV = 2	MNHLVMISLALLLLLGVESVRDAYIAKNYNCVYECFRDAYCNELCTKNGASSGYCQWAGKYGNACWCYALPDNVPIRVPGKCHRK	40	F34; F35; F36	9,571
P45697	Alpha-like toxin BmK-M1 OS = *M. martensii*	MNYLVMISFALLLMTGVESVRDAYIAKPHNCVYECARNEYCNDLCTKNGAKSGYCQWVGKYGNGCWCIELPDNVPIRVPGKCHR	48	F34; F35; F36	9,496
P01480	Alpha-mammal toxin Aah3 OS = *A. australis*	MNYLVMISLALLLMTGVESVRDGYIVDSKNCVYHCVPPCDGLCKKNGAKSGSCGFLIPSGLACWCVALPDNVPIKDPSYKCHSR	12	F35	9068
P09981	Alpha-mammal toxin BeM9 OS = *Mesobuthus eupeus*	ARDAYIAKPHNCVYECYNPKGSYCNDLCTENGAESGYCQILGKYGNACWCIQLPDNVPIRIPGKCH	12	F35	7,343
D5HR50	Alpha-toxin Ac1 (Fragment) OS = *A. crassicauda*	YIVMISLALVVMIGVESVRDGYIVYPNNCVYHCIPACDGLCKKNGGTSGSCSFLIGSGIACWCKDLPDNVPIKDPSQKCTR	16	F31; F33; F34; F35; F38; F46	8,702
P01482	Alpha-toxin Amm5 OS = *A. mauritanicus*	LKDGYIIDDLNCTFFCGRNAYCDDECKKKGGESGYCQWASPYGNACWCYKLPDRVSIKEKGRCN	48	F34; F35; F39; F41	7,301
P01488	Alpha-toxin Bot1 OS = *Buthus occitanus tunetanus*	GRDAYIAQPENCVYECAQNSYCNDLCTKNGATSGYCQWLGKYGNACWCKDLPDNVPIRIPGKCHF	32	F34; F35; F36	7,268
P01489	Alpha-toxin Lqq4 OS = *L. quinquestriatus*	GVRDAYIADDKNCVYTCGSNSYCNTECTKNGAESGYCQWLGKYGNACWCIKLPDKVPIRIPGKCR	86	F35; F36	7,194
P83644	Toxin Lqh4 OS = *L. quinquestriatus hebraeus*	GVRDAYIADDKNCVYTCGANSYCNTECTKNGAESGYCQWFGKYGNACWCIKLPDKVPIRIPGKCR	66	F35; F36	7,211.3
Q9GQW3	Toxin BmKaIT1 OS = *M. martensii*	MNYLVMISFAFLLMTGVESVRDAYIAQNYNCVYHCARDAYCNELCTKNGAKSGSCPYLGEHKFACYCKDLPDNVPIRVPGKCHRR	39	F34; F35; F36	9,649
Q9GYX2	Toxin BmKa1 OS = *M. martensii* PE = 2 SV = 1	MNYLVFFSLALLLMTGVGSVRDGYIADDKNCPYFCGRNAYCDDECKKNGAESGYCQWAGVYGNACWCYKLPDKVPIRVPGKCNGG	25	F34; F35	9,366
Q9GUA7	Toxin BmKa3 OS = *M. martensii* PE = 2 SV = 1	MNYLVFFSLALLLMTGVESVRDGYIADDKNCAYFCGRNAYCDDECKKKGAESGYCQWAGVYGNACWCYKLPDKVPIRVPGKCNGG	25	F34; F35	9,426
Q2YHM1	Neurotoxin 8-related gene product 1/2/3 OS = *A. mauritanicus* PE = 1 SV = 1	VRDAYIAQNYNCVYTCFKNDYCNDICTKNGAXXGYC	78	F34; F35	4,130
P45698	Neurotoxin BmK-M9 OS = *M. martensii* PE = 1 SV = 1	MISFALLLMTGVESVRDAYIAKPENCVYHCATNEGCNKLCTDNGAESGYCQWGGRYGNACWCIKLPDRVPIRVPGKCHR	27	F35; F36	8,756
Q95P69	Toxin BmKT OS = *M. martensii* PE = 2 SV = 1	MNYLVFFSLALLLMTGVESVRDGYIADDKNCAYFCGRNAYCDDECKKNGAESGYCQWAGVYGNACWCYKLPDKVPIRVPGKCNGG	25	F34; F35	9,412
P58328	Alpha-like toxin BmK-M4 OS = *M. martensii*	VRDAYIAKPENCVYHCAGNEGCNKLCTDNGAESGYCQWGGRYGNACWCIKLPDDVPIRVPGKCH	30	F35; F36	7,021
Q9NJC8	Toxin BmKaTx13 OS = *M. martensii*	MNYLVMISFALLLMKGVESVRDAYIAKPENCVYHCAGNEGCNKLCTDNGAESGYCQWGGRYGNACWCIKLPDDVPIRVPGKCHR	23	F35; F36	9,319
Q9N682	Neurotoxin BmK-M11 OS = *M. martensii* PE = 3 SV = 1	MNYLVMISFALLLMTGVESVRDAYIAKPENCVYHCATNEGCNKLCTDNGAESGYCQWGGKYGNACWCIKLPDDVPIRVPGKCHR	22	F35; F36	9,308
D9U2A4	Lipolysis-activating peptide 1-alpha chain OS = *Lychas mucronatus* PE = 2 SV = 1	MNITLFCSVFILISLAGLSVSDDVPGNYPMSLYGNKYSCGVLGENEYCRKICKSHGVSYGYCFNSRCWCEYLEDKDVDFWAAHKNHCKNDKLYPPKK	9	F38; F39	11,094
P01487	Alpha-insect toxin Lqq3 OS = *L. quinquestriatus* PE = 1 SV = 2	VRDAYIAKNYNCVYECFRDSYCNDLCTKNGASSGYCQWAGKYGNACWCYALPDNVPIRVPGKCH	28	F36	7,240
P58488	Alpha-like toxin BmK-M2 OS = *M. martensii* PE = 1 SV = 1	VRDAYIAKPHNCVYECARNEYCNNLCTKNGAKSGYCQWSGKYGNGCWCIELPDNVPIRVPGKCH	36	F34; F35	7,212
P86406	Neurotoxin MeuNaTx-6 OS = *M. eupeus* PE = 1 SV = 1	MMKIIIFLIVSSLVLIGVKTDNGYLLDKYTGCKVWCVINNESCNSECKIRRGNYGYCYFWKLACYCEGAPKSELWHYETNKCNGRM	15	F48; F49	7,785
P55902	Alpha-insect toxin BotIT1 OS = *B. occitanus tunetanus*	VRDAYIAQNYNCVYFCMKDDYCNDLCTKNGASSGYCQWAGKYGNACWCYALPDNVPIRIPGKCHS	75	F34; F35	7,334.93
P01490	Alpha-toxin BeM10 OS = *M. eupeus*	VRDGYIADDKDCAYFCGRNAYCDEECKKGAESGKCWYAGQYGNACWCYKLPDWVPIKQKVSGKCN	14	F34	7,373
P0DJH8	Alpha-toxin Bu1 OS = *Buthacus macrocentrus*	GVRDAYIADDKNCVYTCAKNSYCNTECTKNGAESGYCQWLGKYGNGCWCIKLPDKVPIRIPGRCRGR	75	F35	7,485
P82815	Bukatoxin OS = *M. martensii*	VRDGYIADDKNCAYFCGRNAYCDEECIINGAESGYCQQAGVYGNACWCYKLPDKVPIRVSGECQQ	14	F34	7,226
P15224	Toxin Os1 OS = *Orthochirus scrobiculosus*	ERDGYIVQLHNCVYHCGLNPYCNGLCTKNGATSGSYCQWMTKWGNACYCYALPDKVPIKWLDPKCY	41	F20	7,559
P60256	Toxin Boma6b OS = *Buthus occitanus mardochei*	VRDAYIAQNYNCVYDCARDAYCNDLCTKNGAKSGYCEWFGPHGDACWCIDLPNNVPIKVEGKCHRK	15	F34	7,465
P60258	Toxin Boma6d OS = *B. occitanus mardochei*	VRDAYIAQNYNCVYTCFKDAHCNDLCTKNGASSGYCQWAGKYGNACWCYALPDNVPIRIPGKCHRK	26	F35	7,964
P60259	Toxin Boma6e OS = *B. occitanus mardochei*	VRDAYIAQNYNCVYACARDAYCNDLCTKNGARSGLFATFGPHGDACWCIALPNNVPLKVQGKCHRK	32	F35; F36	7,267
Q9GQV6	Toxin BmKaTx16 OS = *M. martensii*	MNYLVMISFALLLMTGVESVRDAYIAKPHNCVYECARNEYCNDLCTKNGAKSGYCQWVGKYGNGCWCKELPDNVPIRVPGKCHR	48	F34; F35	9,511
M1JBC0	Sodium channel alpha-toxin Acra4 OS = *A. crassicauda* PE = 1 SV = 1	VRDGYIVDDKNCVYHCIPPCDGLCKKNGGKSGSCSFLVPSGLACWCKALPDNVPIKDPSYKCHKR	46	F35	6,937
Q9NJC7	BmK AGP-SYPU2 OS = *M. martensii* PE = 1 SV = 1	MNYMVIISLALLVMTGVESVKDGYIADDRNCPYFCGRNAYCDGECKKNRAESGYCQWASKYGNACWCYKLPDDARIMKPGRCNGG	11	F34	9,524
G4V3T9	Neurotoxin BmK AGAP-SYPU2 (Fragment) OS = *M. martensii* PE = 1 SV = 1	VKDGYIVDDKNCAYFCGRNAYCDDECEKNGAESGYCQWAGVYGNACWCYKLPDKVPIRVPGRCNG	14	F34	7,253
P86404	Neurotoxin MeuNaTx-4 OS = *M. eupeus* PE = 1 SV = 1	MNYLILISFALLVITGVESARDAYIAKPHNCVYECFDAFSSYCNGVCTKNGAKSGYCQILGTYGNGCWCIVLPDNVPIRIPGKCHR	38	F35	9,442
Q17254	Alpha-insect toxin Bot14 OS = *B. occitanus tunetanus* PE = 2 SV = 1	MSSLMISTAMKGKAPYRQVRDGYIAQPHNCAYHCLKISSGCDTLCKENGATSGHCGHKSGHGSACWCKDLPDKVGIIVHGEKCHR	19	F34; F35	9,174
P86405	Neurotoxin MeuNaTx-5 OS = *M. eupeus* PE = 1 SV = 1	MNYLILISFALLVITGVESARDAYIAKPHNCVYECFDAFSSYCNGVCTKNGAKSGYCQILGTYGNGCWCIALPDNVPIRIPGKCHR	38	F35	7,170.47
P13488	Alpha-like toxin Bom3 OS = *B. occitanus mardochei*	GRDGYIAQPENCVYHCFPGSSGCDTLCKEKGATSGHCGFLPGSGVACWCDNLPNKVPIVVGGEKCH	77	F34; F35; F36; F37; F38; F39; F41; F44; F45; F46	6,872
P01485	Alpha-mammal toxin Bot3 (Fragment) OS = *B. occitanus tunetanus*	LVMAGVESVKDGYIVDDRNCTYFCGRNAYCNEECTKLKGESGYCQWASPYGNACYCYKVPDHVRTKGPGRCN	62	F31; F34; F35	8,059
O61705	Neurotoxin BmK-M10 OS = *M. martensii* PE = 1 SV = 1	MNYLIMFSLALLLVIGVESGRDGYIVDSKNCVYHCYPPCDGLCKKNGAKSGSCGFLVPSGLACWCNDLPENVPIKDPSDDCHKR	43	F35; F36; F37; F38; F39; F44; F45	7,040.5
P04099	Alpha-toxin Bot9 OS = *B. occitanus tunetanus*	AEIKVRDGYIVYPNNCVYHCGLNPYCNDLCTKNGAKSGYCQWLTKWGNACYCYALPEKVPIKDPSYKCYS	26	F41	7,998
P56678	Alpha-like toxin Lqh3 OS = *L. quinquestriatus hebraeus* PE = 1 SV = 1	VRDGYIAQPENCVYHCFPGSSGCDTLCKEKGGTSGHCGFKVGHGLACWCNALPDNVGIIVEGEKCHS	43	F35; F37	7,057
P01481	Alpha-mammal toxin Lqq5 OS = *L. quinquestriatus* PE = 1 SV = 1	LKDGYIVDDKNCTFFCGRNAYCNDECKKKGGESGYCQWASPYGNACWCYKLPDRVSIKEKGRCN	14	F34; F35	7,301
Q4TUA4	Alpha-toxin 4 OS = *M. martensii* PE = 1 SV = 1	MNYLVFFSLALLLMTGVESVRDGYIADDKNCAYFCGRNAYCDDECKKKGAESGYCQWAGVYGNACWCYKLPDKVPIRVPGRCNGG	11	F34	7,733.59
P0C910	Alpha-toxin Amm3 OS = *A. mauritanicus* PE = 1 SV = 1	GRDGYIVDTKNCVYHCYPPCDGLCKKNQAKSGSCGFLYPSGLACWCVALPENVPIKDPNDDCHK	53	F35; F36; F38	7,001.82
Q7YXD3	Alpha-toxin Amm8 OS = *A. mauritanicus* PE = 1 SV = 1	MNYLVMISLALLFMTGVESLKDGYIVNDINCTYFCGRNAYCNELCIKLKGESGYCQWASPYGNSCYCYKLPDHVRTKGPGRCNDR	59	F35; F 36	7,382
P01486	Alpha-toxin Bot11 OS = *B. occitanus tunetanus* PE = 1 SV = 1	LKDGYIVDDRNCTYFCGTNAYCNEECVKLKGESGYCQWVGRYGNACWCYKLPDHVRTVQAGRCRS	14	F34	7,468
P59360	Neurotoxin BmK-II OS = *M. martensii* PE = 1 SV = 1	VRDAYIAKPHNCVYECARNEYCNDLCTKDGAKSGYCQWVGKYGNGCWCIELPDNVPIRIPGNCH	47	F35	7,226
Q7Z0H4	Neurotoxin BmP08 OS = *M. martensii* PE = 1 SV = 1	MKIFFAVLVILVLFSMLIWTAYGTPYPVNCKTDRDCVMCGLGISCKNGYCQGCTR	15	F25	6,145
Q7Z0F1	Neurotoxin X-29S OS = *M. martensii* PE = 3 SV = 1	MKIFFAVLVILVLFSMLIWTAYGTPYPVNCKTDRDCVMCGLGISCKNGYCQSCTR	15	F25	6,175
P01479	Neurotoxin-1'' OS = *A. australis* PE = 1 SV = 3	MNYLVMISLALLLMIGVESKRDGYIVYPNNCVYHCVPPCDGLCKKNGGSSGSCSFLVPSGLACWCKDLPDNVPIKDTSRKCTR	29	F31; F33; F34; F35; F36; F38	9,061
M1JMR8	Sodium channel alpha-toxin Acra8 OS = *A. crassicauda* PE = 3 SV = 1	VRDGYIVDDKNCTFFCGRNAYCNDECKKKGGESGYCQWASPYGNACWCYKLPDRVPIKEKGRCNGR	14	F34	7,539
P45658	Toxin Aah4 OS = *A. australis* PE = 1 SV = 2	MNYLIMFSLALLLVIGVESGRDGYIVDSKNCVYHCYPPCDGLCKKNGAKSGSCGFLVPSGLACWCNDLPENVPIKDPSDDCHKR	46	F35; F36; F37; F38; F44; F45	6,882
P21150	Toxin AaHIT4 OS = *A australis* PE = 1 SV = 1	EHGYLLNKYTGCKVWCVINNEECGYLCNKRRGGYYGYCYFWKLACYCQGARKSELWNYKTNKCDL	25	F41	7,786
Q86SE0	Toxin Aam2 OS = *A. amoreuxi* PE = 1 SV = 1	MNYLITISLALLLMTGVASGVRDGYIADAGNCGYTCVANDYCNTECTKNGAESGYCQWFGRYGNACWCIKLPDKVPIKVPGKCNGR	38	F34; F35	9,283
Q9GNG8	Toxin BmKaTX15 OS = *M. martensii* PE = 2 SV = 1	MNYLVFFSLALLVMTGVESVRDGYIADDKNCAYFCGRNAYCDDECKKNGAESGYCQWAGVYGNACWCYKLPDKVPIRVPGKCNGG	25	F34; F35	9,398
P60255	Toxin Boma6a OS = *B. occitanus mardochei* PE = 3 SV = 1	VRDAYIAQNYNCVYDCARDAYCNDLCTKNGAKSGYCEWFGPHGDACWCIDLPNNVPIKVEGKCHRK	32	F35	7,477
P60257	Toxin Boma6c OS = *B. occitanus mardochei* PE = 3 SV = 1	VRDAYIAQNYNCVYTCFKDAHCNDLCTKNGASSGYCQWAGKYGNACWCYALPDNVPIRIPGKCHRK	59	F35	7,440
Q4LCT3	Toxin-like peptide AaF1CA1 OS = *A australis* PE = 2 SV = 1	MMKLVLFSVIVILFSLIGSIHGADVPGNYPLRPFRYRYGCAVPGDSDYCVRVCRKHGVRYGYCWFFTCWCEYLEDKNIKI	11	F38 and F39	9,351
Q9BKJ0	Anti-neuroexcitation peptide 3 OS = *M. martensii* PE = 2 SV = 2	MKLSLLLVISASMLIDGLVNADGYIRGSNGCKISCLWGNEGCNKECKGFGAYYGYCWTWGLACWCEGLPDDKTWKSESNTCGGKK	6	F35	9,271
Q4LCS7	Toxin-like peptide AaF1CA26 OS = *A. australis* GN = aaF1CA26 PE = 2 SV = 1	MMKLMLFSIIVILFSLIGSIHGADVPGNYPLDSSDDTYLCAPLGENPSCIQICRKHGVKYGYCYAFQCWCEYLEDKNVKS	11	F38 and F39	9,019
β-NaScTxs					
P80962	Beta-insect depressant toxin BaIT2 OS = *Buthacus arenicola* PE = 1 SV = 1	DGYIRRRDGCKVSCLFGNEGCDKECKAYGGSYGYCWTWGLACWCEGLPDDKTWKSETNTCG	20	F36; F44	6,845
Q17230	Beta-insect depressant toxin BmKIT4 OS = *M. martensii* PE = 2 SV = 2	DGYIRGSNGCKISCLWGNEGCNKECKGFGAYYGYCWTWGLACWCEGLPDDKTWKSESNTCGRKK	8	F35	7,186
Q9XY87	Beta-insect depressant toxin BmKITa OS = *M. martensii* PE = 1 SV = 1	MKLFLLLLISASMLIDGLVNADGYIRGSNGCKVSCLWGNEGCNKECRAYGASYGYCWTWGLACWCQGLPDDKTWKSESNTCGGKK	12	F36; F38	9,312
Q95WX6	Beta-insect depressant toxin BmKITb OS = *M. martensii* PE = 1 SV = 1	MKLFLLLVISASMLIDGLVNADGYIRGSNGCKVSCLWGNEGCNKECKAFGAYYGYCWTWGLACWCQGLPDDKTWKSESNTCGGKK	12	F35; F36; F38	9,330
P55903	Beta-insect depressant toxin BotIT4 OS = *B. occitanus tunetanus* PE = 1 SV = 1	DGYIRRRDGCKVSCLFGNEGCDKECKAYGGSYGYCWTWGLACWCEGLPDDKTWKSETNTCG	20	F37; F44; F45	6,836.25
P55904	Beta-insect depressant toxin BotIT5 OS = *B. occitanus tunetanus* PE = 1 SV = 1	DGYIRKRDGCKVSCLFGNEGCDKECKAYGGSYGYCWTWGLACWCEGLPDDKTWKSETNTCG	20	F37; F44; F45	6,791.78
Q26292	Beta-insect depressant toxin LqhIT2 OS = *L. quinquestriatus hebraeus* PE = 1 SV = 1	MKLLLLLIVSASMLIESLVNADGYIKRRDGCKVACLIGNEGCDKECKAYGGSYGYCWTWGLACWCEGLPDDKTWKSETNTCGGKK	33	F34; F35	9,334
O77091	Beta-insect excitatory toxin BmK IT-AP OS = *M. martensii* GN = IT-AP PE = 1 SV = 1	MKFFLIFLVIFPIMGVLGKKNGYAVDSSGKVAECLFNNYCNNECTKVYYADKGYCCLLKCYCFGLADDKPVLDIWDSTKNYCDVQIIDLS	20	F41	8,149.39
P68721	Beta-insect excitatory toxin LqhIT1a OS = *L. quinquestriatus hebraeus* PE = 3 SV = 1	MKFFLLFLVVLPIMGVLGKKNGYAVDSKGKAPECFLSNYCNNECTKVHYADKGYCCLLSCYCFGLNDDKKVLEISGTTKKYCDFTIIN	10	F39; F41	9,901
P68722	Beta-insect excitatory toxin LqhIT1b OS = *L. quinquestriatus hebraeus* PE = 1 SV = 1	MKFFLLFLVVLPIMGVLGKKNGYAVDSKGKAPECFLSNYCNNECTKVHYADKGYCCLLSCYCFGLNDDKKVLEISDTTKKYCDFTIIN	10	F39; F41	9,959
P68723	Beta-insect excitatory toxin LqhIT1c OS = *L. quinquestriatus hebraeus* PE = 1 SV = 1	MKFFLLFLVVLPIMGVLGKKNGYAVDSKGKAPECFFSNYCNNECTKVHYAEKGYCCLLSCYCVGLNDDKKVMEISDTRKKICDTTIIN	18	F38; F39; F41	9,936
P0C5H3	Beta-mammal/insect toxin Lqhb1 OS = *L. quinquestriatus hebraeus* PE = 1 SV = 1	MKIIIFLIVSSLMLIGVKTDNGYLLNKATGCKVWCVINNASCNSECKLRRGNYGYCYFWKLACYCEGAPKSELWAYATNKCNGKL	12	F48; F49	9,574
Q9UAC8	Beta-toxin BmKAs1 OS = *M. martensii* PE = 1 SV = 1	MKIIIFLIVCSFVLIGVKADNGYLLNKYTGCKIWCVINNESCNSECKLRRGNYGYCYFWKLACYCEGAPKSELWAYETNKCNGKM	12	F48; F49	7,721
P59863	Beta-toxin BotIT2 OS = *B. occitanus tunetanus* PE = 1 SV = 1	DGYIKGYKGCKITCVINDDYCDTECKAEGGTYGYCWKWGLACWCEDLPDEKRWKSETNTC	18	F34; F35; F38; F44	6,918
Q4LCT0	Beta-toxin KAaH1 OS = *A. australis* PE = 1 SV = 1	MMKLMLFSIIVILFSLIGSIHGADVPGNYPLDSSDDTYLCAPLGENPFCIKICRKHGVKYGYCYAFQCWCEYLEDKNVKI	11	F38; F39	6,652.15
Q4LCS9	Beta-toxin KAaH2 OS = *A. australis* PE = 1 SV = 1	MMKLMLFSIIVILFSLIGSIHGADVPGNYPLDSSDDTYLCAPLGENPSCIQICRKHGVKYGYCYAFQCWCEYLEDKNVKI	11	F38; F39	6,592.5
P68725	Insect toxin 2-13 OS = *L. quinquestriatus hebraeus* PE = 1 SV = 1	MKLLLLLIITASMLIEGLVNADVYIRRHDGCKISCTVNDKYCDNECKSEGGSYGYCYAFGCWCEGLPNDKAWKSETNTCGGKK	12	F34; F35	9,211
P68726	Insect toxin 2-53 OS = *L. quinquestriatus hebraeus* PE = 1 SV = 1	MKLLLLLIVSASMLIESLVNADGYIKRRDGCKVACLVGNEGCDKECKAYGGSYGYCWTWGLACWCEGLPDDKTWKSETNTCGGKK	33	F34; F35	9,320
P81504	Insect toxin AaHIT5 OS = *A. australis* PE = 1 SV = 1	DGYIKRHDGCKVTCLINDNYCDTECKREGGSYGYCYSVGFACWCEGLPDDKAWKSETNTCD	39	F34; F35	6,882
P82812	Insect toxin BsIT2 OS = *Hottentotta tamulus sindicus* PE = 1 SV = 1	DGYIKKSKGCKVSCVINNVYCNSMCKSLGGSYGYCWTYGLACWCEGLPNAKRWKYETKTCK	8	F44	6,892.4
P80950	Neurotoxin-like protein STR1 OS = *A. australis* PE = 1 SV = 1	ARDGYIVHDGTNCKYSCEFGSEYKYCGPLCEKKKAKTGYCYLFACWCIEVPDEVRVWGEDGFMCWS	38	F38; F39; F41	7,641
P15228	Toxin BmKAEP OS = *M. martensii* PE = 1 SV = 2	MKLFLLLVISASMLIDGLVNADGYIRGSNGCKVSCLLGNEGCNKECRAYGASYGYCWTWKLACWCQGLPDDKTWKSESNTCGGKK	12	F36; F38	9,296
P86408	Neurotoxin MeuNaTx-1 OS = *M. eupeus* PE = 1 SV = 1	MNSLVMISLALLVMTGVESVRDGYIADDKNCAYFCGRNAYCDEECKKKGAESGYCQWAGQYGNACWCYKLPDKVPIKVSGKCNGR	14	F34	7,178.27
E7BLC7	Toxin Acra3 OS = *A. crassicauda* PE = 1 SV = 1	MKIIFLVLMMILSEVYSDRDGYPVHDGTNCKYSCDIREKWEYCTPLCKRRNAKTGYCYAFACWCIGLPDEVKVYGDDGIFCKSG	17	F39	7,620.3

The analysis of the different fractions of interest allowed the identification of 42 peptides corresponding to KScTxs, with a sequence coverage ranging from 13% (K7XFK5) to 100% (P56215), 33 are those belonging to the alpha family ‘α-KScTxs’, while nine were corresponding to beta family β-KScTxs (Table 4).

**TABLE 4:** The different KScTxs identified in the fractions of *A. mauritanicus* venom.

Accession number	Name	Sequence	Coverage (%)	Fractions	MW (Da)
KScTxs					
α-KScTxs					
P60233	Potassium channel toxin alpha-KTx 15.1 OS = *A. australis* PE = 1 SV = 1	QNETNKKCQGGSCASVCRRVIGVAAGKCINGRCVCYP	81	F29	3,875
P60208	Potassium channel toxin alpha-KTx 15.3 OS = *A. mauritanicus* PE = 1 SV = 1	QNETNKKCQGGSCASVCRRVIGVAAGKCINGRCVCYP	84	F29	3,823.5
Q867F4	Potassium channel toxin alpha-KTx 15.4 OS = *A. australis* PE = 1 SV = 1	MKFSSIILLTLLICSMSIFGNCQIETNKKCQGGSCASVCRRVIGVAAGKCINGRCVCYP	51	F29	6,291
Q86SD8	Potassium channel toxin alpha-KTx 15.5 OS = *A. australis* PE = 2 SV = 1	MKFSSIILLTLLICSMSIFGNCQVETNKKCQGGSCASVCRRVIGVAAGKCINGRCVCYP	51	F29	6,277
Q5K0E0	Potassium channel toxin alpha-KTx 15.7 OS = *A. amoreuxi* PE = 1 SV = 1	MKFSSIILLTLLICSMSIFGNGQVQTNKKCKGGSCASVCAKEIGVAAGKCINGRCVCYP	36	F29	3,751.48
B8XH42	Potassium channel toxin alpha-KTx 16.6 OS = *Buthus occitanus israelis* PE = 2 SV = 1	MKILSVLLIALIICSINICSEAGLIDVRCYASRECWEPCRRVTGSAQAKCQNNQCRCY	19	F25	6,512
P0DL46	Potassium channel toxin alpha-KTx 16.9 OS = *Buthus paris* PE = 1 SV = 1	GLIDVRCYASRECWEPCRKVTGSGQAKCQNNQCRCY	75	F25	4,121
Q95NJ8	Potassium channel toxin alpha-KTx 17.1 OS = *M. martensii* PE = 1 SV = 1	MKFIIVLILISVLIATIVPVNEAQTQCQSVRDCQQYCLTPDRCSYGTCYCKTTGK	16	F25	6,161
B8XH44	Potassium channel toxin alpha-KTx 27.1 OS = *B. occitanus israelis* PE = 3 SV = 1	MKFLFLTLFVCCFIAVLVIPSEAQIDINVSCRYGSDCAEPCKRLKCLLPSKCINGKCTCYPSIKIKNCKVQTY	34	F25; F28; F34; F31; F 33	8,218
P24662	Potassium channel toxin alpha-KTx 3.1 OS = *A. mauritanicus* PE = 1 SV = 2	GVEINVKCSGSPQCLKPCKDAGMRFGKCMNRKCHCTPK	32	F29; F31; F34	4,156
P0C909	Potassium channel toxin alpha-KTx 3.11 OS = *Odontobuthus doriae* PE = 1 SV = 1	GVPTDVKCRGSPQCIQPCKDAGMRFGKCMNGKCHCTPK	21	F29; F31; F34	4,072
P0C8R1	Potassium channel toxin alpha-KTx 3.12 OS = *A. amoreuxi* PE = 1 SV = 1	VGINVKCKHSGQCLKPCKDAGMRFGKCMNGKCDCTPK	21	F29; F31; F34	4,184.08
P86396	Potassium channel toxin alpha-KTx 3.13 OS = *M. eupeus* PE = 1 SV = 1	VGINVKCKHSGQCLKPCKDAGMRFGKCMNGKCDCTPK	22	F29; F31; F34	3,980.8
K7XFK5	Potassium channel toxin alpha-KTx 3.16 OS = *Mesobuthus gibbosus* PE = 2 SV = 1	MKVFSAVLIILFVCSMIIGISEGKEIPVKCKHSGQCLQPCKDAGMRFGKCMNGKCNCTPK	13	F29; F31; F34	6,523
C0HJQ6	Potassium channel toxin alpha-KTx 3.19 OS = *M. eupeus* PE = 1 SV = 1	VGINVKCKHSGQCLKPCKDAGMRFGKCINGKCDCTPK	22	F29; F31; F34	3,962
P31719	Potassium channel toxin alpha-KTx 5.2 OS = *A. mauritanicus* PE = 1 SV = 1	GVPINVSCTGSPQCIKPCKDAGMRFGKCMNRKCHCTPK	22	F29; F31; F34	4,097
P46112	Potassium channel toxin alpha-KTx 3.3 OS = *L. quinquestriatus hebraeus* PE = 1 SV = 1	GVPINVPCTGSPQCIKPCKDAGMRFGKCMNRKCHCTPK	21	F29; F31; F34	4,107
P46110	Potassium channel toxin alpha-KTx 3.4 OS = *L. quinquestriatus hebraeus* PE = 1 SV = 1	GVPINVKCTGSPQCLKPCKDAGMRFGKCINGKCHCTPK	21	F29; F31; F34	4,021
P45696	Potassium channel toxin alpha-KTx 3.5 OS = *A. australis* GN = KTX2 PE = 1 SV = 1	MKVFSAVLIILFVCSMIIGINAVRIPVSCKHSGQCLKPCKDAGMRFGKCMNGKCDCTPK	14	F29; F31; F34	6,393
Q9NII7	Potassium channel toxin alpha-KTx 3.6 OS = *M. martensii* PE = 1 SV = 1	MKVFFAVLITLFICSMIIGIHGVGINVKCKHSGQCLKPCKDAGMRFGKCINGKCDCTPKG	13	F29; F31; F34	6,462
P59886	Potassium channel toxin alpha-KTx 3.8 OS = *H. tamulus sindicus* PE = 1 SV = 1	GVPINVKCRGSPQCIQPCRDAGMRFGKCMNGKCHCTPQ	21	F29; F31; F34	4,121.4
P59290	Potassium channel toxin alpha-KTx 3.9 OS = *B. occitanus tunetanus* GN = KTX3 PE = 1 SV = 1	VGIPVSCKHSGQCIKPCKDAGMRFGKCMNRKCDCTPK	43	F25; F29; F31; F34	4,376.27
P56215	Potassium channel toxin alpha-KTx 8.1 OS = *Androctonus mauritanicus* PE = 1 SV = 1	VSCEDCPEHCSTQKAQAKCDNDKCVCEPI	100	F19; F21; F22	3,185
P80671	Potassium channel toxin alpha-KTx 8.4 OS = *L. quinquestriatus hebraeus* PE = 1 SV = 1	VSCEDCPDHCSTQKARAKCDNDKCVCEPK	79	F19; F20; F22	3,214
P0CC12	Potassium channel toxin alpha-KTx 8.5 OS = *O. doriae* PE = 1 SV = 1	VSCEDCPEHCSTQKARAKCDNDKCVCESV	48	F19; F22	3,180.12
A0A1L2FZD4	Potassium channel toxin alpha-KTx 8.8 OS = *O. scrobiculosus* GN = OSK3 PE = 1 SV = 1	MCRLYAIILIVLVMNVIMTIIPDSKVEVVSCEDCPEHCSTQKARAKCDNDKCVCEPI	44	F19; F21; F22	3,206.3
Q9NJP7	Potassium channel toxin alpha-KTx 9.1 OS = *M. martensii* PE = 1 SV = 1	MSRLFTLVLIVLAMNVMMAIISDPVVEAVGCEECPMHCKGKNAKPTCDDGVCNCNV	48	F19; F25; F28	2,950.72
Q9U8D1	Potassium channel toxin alpha-KTx 9.2 OS = *M. martensii* PE = 1 SV = 1	MSRLFTLVLIVLAMNVMMAIISDPVVEAVGCEECPMHCKGKNANPTCDDGVCNCNV	50	F25; F28	2,935.29
P80669	Potassium channel toxin alpha-KTx 9.3 OS = *L. quinquestriatus hebraeus* PE = 1 SV = 1	VGCEECPMHCKGKNAKPTCDNGVCNCNV	96	F19; F 22	2,954
P84744	Potassium channel toxin alpha-KTx 9.5 OS = *B. occitanus tunetanus* PE = 1 SV = 1	VGCEECPMHCKGKHAVPTCDDGVCNCNV	43	F28	2,942.58
P83406	Potassium channel toxin alpha-KTx 9.7 OS = *Hottentotta judaicus* PE = 1 SV = 1	VGCEECPAHCKGKNAIPTCDDGVCNCNV	61	F22; F25	2,874.46
B8XH46	Potassium channel toxin alpha-KTx 9.8 OS = *B. occitanus israelis* PE = 2 SV = 1	MSRLFTLVLIVLAMNVMMAIISDPVVEAVGCEECPMHCKGKMAKPTCDDGVCNCNV	48	F19; F25; F28	6,032
B8XH33	Potassium channel toxin alpha-KTx 9.9 (Fragment) OS = *B. occitanus israelis* PE = 2 SV = 2	KKTSRLFTLVLIVLAMNVMMAIISDPVVEAVGCEECPMHCKGKMAKPTCYDGVCNCNV	21	F28	6,307
β-KScTxs					
Q9NJC6	Potassium channel toxin BmTXK-beta OS = *M. martensii* PE = 2 SV = 1	MMKQQFFLFLAVIVMISSVIEAGRGKEIMKNIKEKLTEVKDKMKHSWNKLTSMSEYACPVIEKWCEDHCAAKKAIGKCEDTECKCLKLRK	31	F28; F29; F31; F 33; F 34; F 35; F 36; F 37; F 38; F 39; F 44	10,431
Q9N661	Potassium channel toxin BmTXK-beta-2 OS = *M. martensii* PE = 2 SV = 1	MQRNLVVLLFLGMVALSSCGLREKHFQKLVKYAVPEGTLRTIIQTAVHKLGKTQFGCPAYQGYCDDHCQDIKKEEGFCHGFKCKCGIPMGF	56	F19; F 20; F 25; F28; F 33; F35; F 36; F 41; F44; F45; F48	10,212
B8XH40	Potassium channel toxin BuTXK-beta OS = *B. occitanus israelis* PE = 2 SV = 1	MQRNLVVLLLLGMVALSSCGLREKHFQKLVKYAVPESTLRTILQTAVHKLGKTQFGCPAYQGYCDDHCQDIKKEEGFCHGMKCKCGIPMGF	43	F28; F 33	10,192
A0A059UI30	Potassium channel toxin Meg-beta-KTx1 OS = *M. gibbosus* PE = 3 SV = 1	MQRNLVVLLFLGMVALSSCGLREKHFQKLVKYAVPEGTLRTIIQTAVHKLGKTQFGCPAYQGYCDDHCQDIKKQEGFCHGFKCKCGIPMGF	38	F19; F20; F25; F28; F31; F33; F35; F36; F37; F 41; F44; F45; F 48	10,211
P0CH57	Potassium channel toxin MeuTXKbeta3 OS = *M. eupeus* PE = 1 SV = 1	MMKQQFFLFLAVIVMISSVIEAGRGREFMSNLKEKLSGVKEKMKNSWNRLTSMSEYACPVIEKWCEDHCQAKNAIGRCENTECKCLSK	27	F29; F34; F36; F37; F38; F44	10,144
P69939	Potassium channel toxin AaTXK-beta OS = *A. australis* PE = 1 SV = 1	MQRNLVVLLFLGMVALSSCGLREKHVQKLVKYAVPVGTLRTILQTVVHKVGKTQFGCPAYQGYCDDHCQDIKKEEGFCHGFKCKCGIPMGF	35	F29	10,148
P15230	Peptide 2 OS = *H. tamulus sindicus* PE = 1 SV = 1	VGCEEDPMHCKGKQAKPTCCNGVCNCNV	56	F54	2,968
P86399	Neurotoxin lambda-MeuTx OS = *M. eupeus* PE = 1 SV = 2	MSTFIVVFLLLTAILCHAEHAIDETARGCNRLNKKCNSDADCCRYGERCISTGVNYYCRPDFGP	25	F28; F29; F35	7,177
P80670	Gating modifier of anion channels 2 OS = *L. quinquestriatus hebraeus* PE = 1 SV = 1	VSCEDCPDHCSTQKARAKCDN DKCVCEPI	79	F19	3,191.29

Five peptides corresponding to ClScTx were detected with a sequence coverage of 43% (Insectotoxin-I5; P60270) at 100% Neurotoxin P2; P01498) (Table 5).

**TABLE 5:** The different ClScTxs identified in the fractions of *A. mauritanicus* venom.

Accession number	Name	Sequence	Coverage (%)	Fractions	MW
P45639	Chlorotoxin OS = *L. quinquestriatus*	MCMPCFTTDHQMARKCDDCCGGKGRGKCYGPQCLCR	75	F34; F35; F36; F37; F38; F39; F41; F44; F45; F46	4,005
P86436	Chlorotoxin-like peptide OS = *A. australis*	MCIPCFTTNPNMAAKCNACCGSRRGSCRGPQCIC	53	F28; F29; F31; F33; F34; F 35; F36; F38; F39	3,607.8
P60270	Insectotoxin-I5 OS = *M. eupeus*	MCMPCFTTDPNMANKCRDCCGGGKKCFGPQCLCNR	43	F35	3,835
Q9UAD0	Neurotoxin BmK CT OS = *M. martensii*	MKFLYGIVFIALFLTVMFATQTDGCGPCFTTDANMARKCRECCGGIGKCFGPQCLCNRI	64	F31	3,747.48
P01498	Neurotoxin P2 OS = *A. mauritanicus*	CGPCFTTDPYTESKCATCCGGRGKCVGPQCLCNRI	100	F28; F31; F33; F34; F35; F36; F37; F38; F39; F41; F44; F45; F46	3,673

Interestingly, among the identified neurotoxins, one peptide shares a similarity of 25% with the toxin BmCa-1 (Q8I6X9). A CaScTx was identified for the first time in *Mesobuthus martensii* (Table 6).

**TABLE 6:** The CaScTx identified in the fractions of *A. mauritanicus* venom.

Accession number	Name	Sequence	Coverage (%)	Fractions	MW
Q8I6X9	Toxin BmCa-1 OS = *M. martensii*	MNTFVVVFLLLTAILCHAEHALDETARGCNRLNKKCNSDGDCCRYGERCISTGVNYYCRPDFGP	25	F28; F29	7,176

Moreover, other than neurotoxins, we identified other peptides generally with a low sequence coverage (Table 7) corresponding to:

- AMPs: (Venom antimicrobial peptide (E4VP07); Antimicrobial peptide 1 (G8YYA5) and Antimicrobial peptide 2 (G8YYA6);
- Amphipathic peptides (Amphipathic peptide Tx348 (B8XH50); Mauriporin (N0EAL3) and Bradykinin-potentiating peptide NDBP6 (D9U2B5).

**TABLE 7:** Other peptides identified in the fractions of *A. mauritanicus* venom.

Accession number	Name	Sequence	Coverage (%)	Fractions	MW
Antimicrobial peptides (AMPs)					
Q9GQW4	Peptide BmKn1 OS = *M. martensii*	MKSQTFFLLFLVVLLLAISQSEAFIGAVAGLLSKIFGKRSMRDMDTMKYLYDPSLSAADLKTLQKLMENY	20	F35; F39; F45; F46	7,913
Q6JQN2	Peptide BmKn2 OS = *M. martensii*	MKSQTFFLLFLVVLLLAISQSEAFIGAIANLLSKIFGKRSMRDMDTMKYLYDPSLSAADLKTLQKLMENY	19	F35; F39; F45; F46	7,985
E4VP07	Venom antimicrobial peptide-6 OS = *M. eupeus*	MKSQTFFLLFLVVFLLAITQSEAIFGAIAGLLKNIFGKRSLRDMDTMKYLYDPSLSAADLKTLQKLMENY	19	F35; F39; F45; F46	7,985
G8YYA5	*Antimicrobial peptide* 1 OS = *A. amoreuxi*	MEIKYLLTVFLVLLIGSDYCQAFLFSLIPHAIGGLISAFKGRRKRDLDGQIDRSRNFRKRDAELEELLSKLPIY	16	F33	1,931.94
G8YYA6	*Antimicrobial peptide* 2 OS = *A. amoreuxi*	MEIKYLLTVFLVLLIVSDHCQAFPFSLIPHAIGGLISAIKGRRKRDLDGQIDRSRNFRKRDAELEELLSKLPIY	16	F33	1,880.93
Amphipathic peptides					
B8XH50	Amphipathic peptide Tx348 OS = *B. occitanus israelis*	MKSQAFFLLFLVVLLLATTQSEAFIMDLLGKIFGRRSMRNMDTMKYLYDPSLSAADLKTLQKLMENY	19	F35; F39; F45; F46	7,773
N0EAL3	Mauriporin OS = *A. mauritanicus* PE = 1 SV = 1	MNKKTLLVIFFITMLIVDEVNSFKIGGFIKKLWRSKLAKKLRAKGRELLKDYANRVINGGPEEEAAVPAERRR	38	F28; F29; F31; F33; F34	5,398.1
D9U2B5	Bradykinin-potentiating peptide NDBP6 OS = *L. mucronatus* PE = 1 SV = 1	MNKKTLLVIFFVTMLIVDEVNSFRFGSFLKKVWKSKLAKKLRSKGKQLLKDYANRVLNGPEEEAAAPAERRR	18	F 21; F28; F29	8,355

**FIGURE 3:**
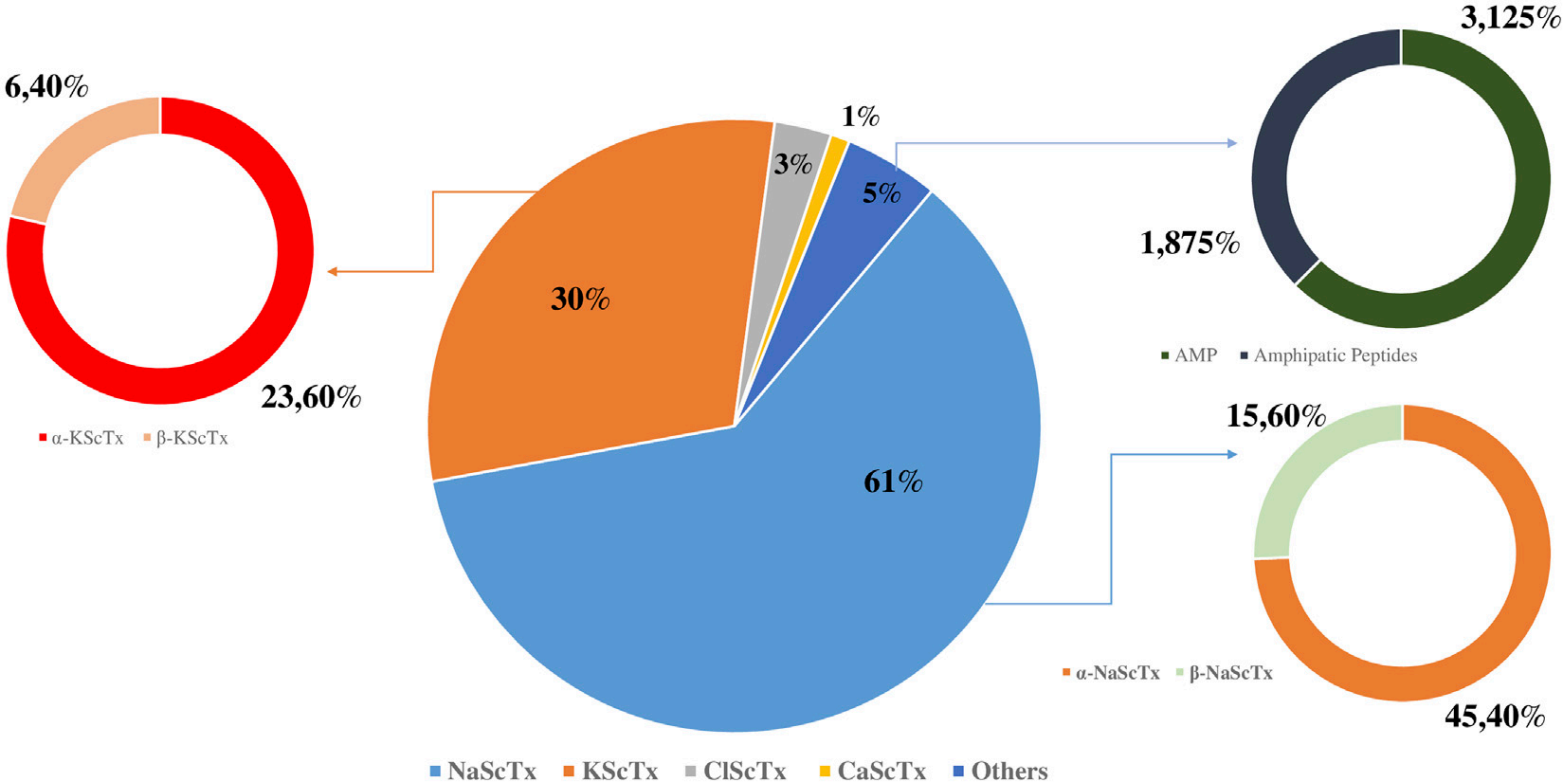
Proportional composition of peptides in *Androctonus mauritanicus* venom.

To provide a global overview, the proportional distribution of these toxin families presents in *A. Mauritanicus* venom is illustrated in Figure 3 highlighting the predominance of NaScTxs, followed by KScTxs, while CaScTxs, ClScTxs and other peptides were less abundant.

### 3.4 ELISA

**FIGURE 4:**
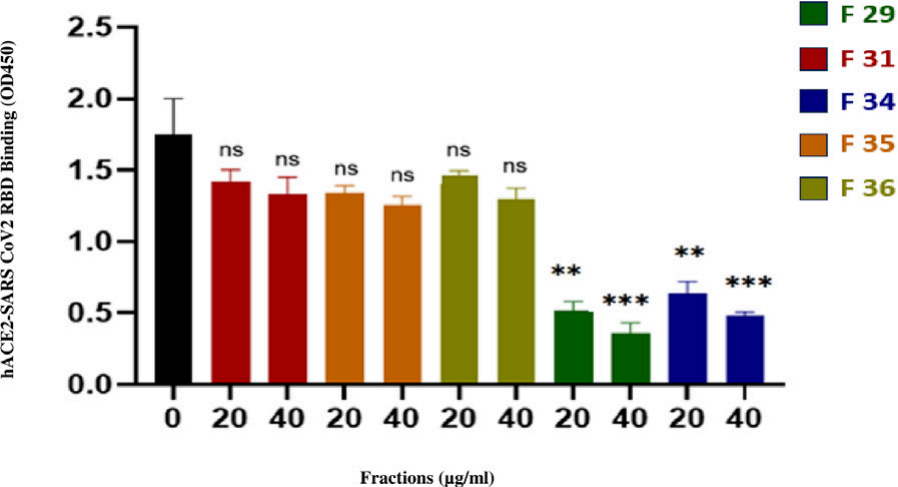
Inhibition potency of fractions on the interaction between ACE2 and SARS-CoV-2 spike (RBD) protein.

The enzyme-linked immunosorbent assay was employed to evaluate the inhibitory effects of various fractions on the interaction between ACE2 and the receptor-binding domain (RBD) of the SARS-CoV-2 Spike protein. As summarized in Table 8 and illustrated in Figure 4, fractions F29 and F34 demonstrated the strongest inhibitory activity, with 79.7% and 73.9% inhibition at 40 μg/mL, respectively. Both fractions exhibited distinct retention times (42 min; 49 min) and had estimated IC_50_ values below 20 μg/mL, indicating high potency even at low concentrations.

**TABLE 8:** Inhibition of ACE2-RBD interaction by fractions at different concentrations.

Fraction	Retention time (min)	Concentration (µg/mL)	OD450	Inhibition (%)	Estimated IC50 (µg/mL)
F29	42	20	0.50	71.01	<20
F29	42	40	0.35	79.71	<20
F31	45	20	1.45	15.94	>40
F31	45	40	1.31	23.91	>40
F34	49	20	0.65	62.32	<20
F34	49	40	0.45	73.91	<20
F35	51	20	1.33	23.19	>40
F35	51	40	1.24	28.12	>40
F36	52	20	1.45	15.94	>40
F36	52	40	1.28	26.09	>40

Fractions F31, F35, and F36 displayed lower inhibition (15.9%–28.1% at 40 μg/mL) and correspondingly higher IC_50_ values (>40 μg/mL), suggesting a moderate effect on ACE2-RBD interaction. The negative control showed no inhibition, confirming the specificity of the assay. These results highlight the differences in inhibitory potency among the tested fractions and suggest that F29 and F34 are promising candidates for further therapeutic exploration.

### 3.5 *In Vivo* neurotoxicity evaluation of *A. mauritanicus* venom fractions

The neurotoxic potential of *A. mauritanicus* crude venom and selected RP-HPLC fractions was evaluated *in vivo* using intracerebroventricular (ICV) injections in Swiss mice. Clinical signs, mortality within 2 h post-injection, and LD_50_ values were recorded where applicable. The results are summarized in Table 9.

**TABLE 9:** Neurotoxicity profile of crude venom and selected RP-HPLC fractions of *A. mauritanicus*.

Venom/Fraction	Toxicity	Mortality after 2 h	LD_50_ (µg/g)
Crude venom	+++	Yes	2.40
Fraction 29 (F29)	–	No	
Fraction 34 (F34)	–	No	–
Fraction 31 (F31)	+	Yes	1.21
Fraction 35 (F35)	–	No	–
Fraction 36 (F36)	–	No	–
F29 + F34	–	No	–

Fractions 29 and 34, despite demonstrating high Spike–ACE2 inhibition in ELISA, did not induce any observable toxicity or mortality, even when combined, indicating excellent safety profiles *in vivo*. In contrast, fraction 31 showed mild neurotoxicity with delayed onset of symptoms and resulted in mortality at higher doses, with an estimated LD_50_ of 1.21 μg/g. Fractions 35 and 36 were inactive in both antiviral and neurotoxicity assays, showing no behavioral effects or lethality.

## 4 Discussion

The venom of *A. mauritanicus* is a complex mixture containing peptides, enzymes, and small proteins, many of which exhibit neurotoxic properties (Watt and Simard, 1984). These molecules target ion channels, such as sodium, potassium, and calcium channels, interfering with the nervous system of prey or potential predators (Quintero-Hernández et al., 2013). However, beyond these toxic effects, the venom contains bioactive peptides that possess antimicrobial, antifungal, and antiviral activities (Hong et al., 2014; Harrison et al., 2014; Guilhelmelli et al., 2016). The molecular diversity of these peptides makes *A. mauritanicus* venom a promising candidate for bioprospecting, particularly in the search for new drugs (Xia et al., 2023; Hilal et al., 2023b). In the context of SARS-CoV-2, which causes COVID-19, peptides from *A. mauritanicus* venom could offer a novel antiviral strategy. Proteins like the SARS-CoV-2 spike protein (S protein) are crucial for viral entry, making them attractive targets for inhibition. The discovery and characterization of venom peptides capable of binding to or blocking the S protein could provide valuable leads for the development of new antiviral drugs (Ghazal et al., 2024).

This study aimed to fractionate *A. mauritanicus* venom using Reverse Phase High-Performance Liquid Chromatography (RP-HPLC) and characterize the resulting fractions via various mass spectrometry platforms, including triple quadrupole ESI-MS, Q-TOF LC/MS, and Q-Exactive LC/MS. The primary objective was to identify novel neurotoxins and isolate those responsible for severe symptoms, as well as those with potential therapeutic or biotechnological applications. The RP-HPLC fractionation of *A. mauritanicus* venom yielded 80 fractions (Figure 2). A comparable number of fractions were identified in the venom of *Opisthacanthus cayaporum*, while fewer fractions were obtained from other species: *Buthacus macrocentrus* (70 fractions), *Tityus pachyurus* (57 fractions), *M. martensii* (29 fractions), and *Heterometrus longimanus* (19 fractions) (Schwartz et al., 2008; Caliskan et al., 2012; Barona et al., 2006; Xu et al., 2014; Bringans et al., 2008). Each fraction was analyzed using direct infusion into the ESI-MS triple quadrupole spectrometer to determine average molecular masses, providing insights into the proteome of each fraction (Volmer et al., 2007). The analysis yielded 507 distinct molecular masses (Table 2), surpassing the number obtained from whole-venom analysis, highlighting the value of RP-HPLC for enhancing molecular resolution (Kumar and Kumar, 2012).

Fractions of interest were further analyzed using Q-TOF LC/MS to generate monoisotopic masses. The results confirmed significant heterogeneity in the composition of individual fractions. Differences in the number of masses detected by ESI-MS and Q-TOF LC/MS reflected the enhanced sensitivity of Q-TOF LC/MS for monoisotopic mass detection, coupled with nano-HPLC separation. The molecular diversity of the fractions was evident, with each containing multiple bioactive peptides with diverse biological properties. Fractions F19, F20, F21, F22, F25, F28, and F29 were found to be enriched in peptides corresponding to neurotoxins such as KScTxs, ClScTxs, and CaScTxs (Table 3–6). Notably, NaScTxs were detected only from fraction F31 onward, albeit with limited sequence coverage.

In total, 86 NaScTx-related peptides were identified, with sequence coverage ranging from 9% (Lipolysis-activating peptide 1-alpha chain, D9U2A4) to 86% (Alpha-toxin Lqq4, P01489). Additionally, 42 KScTx peptides were detected, with sequence coverage spanning from 13% (Alpha-KTx 3.16, K7XFK5) to 100% (Alpha-KTx 8.1, P56215). Among the ClScTxs, five peptides were identified with sequence coverage between 43% (Insectotoxin-I5, P60270) and 100% (Neurotoxin P2, P01498). A single CaScTx-related peptide was detected, exhibiting 25% sequence similarity to Toxin BmCa-1 (Q8I6X9).

Interestingly, 55 NaScTx peptides were identified for the first time, with 31 matching those reported in previous studies on *A. mauritanicus* venom. Fragments of some of the most toxic NaScTxs were detected, including:

- Alpha-toxin Amm 5 in fractions F34, F35, F39, and F41, with the highest sequence coverage (48%) observed in fraction F41.
- Alpha-toxin Amm 3 in fractions F35, F36, and F38, with maximum coverage (52%) in fractions F35 and F36.
- Alpha-mammal toxin Lqq5 in fractions F34 and F35, with 14% sequence coverage.

These neurotoxins were exclusively detected by Q-Exactive LC/MS and not by Q-TOF LC/MS, likely due to the superior sensitivity of the Orbitrap analyzer used in Q-Exactive (Fedorova et al., 2013). This instrument’s High-Energy Collisional Dissociation (HCD) chamber provides higher collision energy than the Collision-Induced Dissociation (CID) mechanism used in Q-TOF LC/MS, enabling improved peptide fragmentation. However, both CID and HCD were limited in efficiently fragmenting long toxins, resulting in low sequence coverage for intact neurotoxins.

Regarding KScTx, 32 peptides were identified, 10 of which had previously been detected. The analysis revealed that α-KScTxs exhibited notable molecular diversity, with seven subfamilies identified: Alpha-KTx 03, 08, 09, 15, 16, 17, and 27. Some KScTx peptides displayed sequence homology with:

- Gating modifiers of anion channels (P80670), potent inhibitors of chloride channel ClC-2/CLCN2.
- Alpha-KTx 9.1 homolog (Q9NJP7), a specific inhibitor of small-conductance potassium channels (KCa2).
- Alpha-KTx 9.7 homolog (P83406), an activator of calcium channels that reversibly modulates the ryanodine receptor 1 (RYR1).

Only α-KScTxs and β-KScTxs were identified in this study, while other scorpion venoms, such as that of *Centruroides hirsutipalpus,* are known to contain additional KScTx families (γ-, δ-, and ε-) (Table 4) (Valdez-Velázquez et al., 2020).

Two chloride channel-targeting neurotoxins were also identified:

- A chlorotoxin-like peptide with a molecular mass of 3624.28 Da in fraction F33% and 53% sequence coverage.
- Chlorotoxin with a monoisotopic mass of 3806.45 Da in fraction F44% and 42% sequence coverage.

For CaScTxs, only one peptide was detected, with 25% sequence homology to BmCa-1 (Q8I6X9) (Table 6). This result aligns with previous findings that CaScTxs are relatively rare in scorpion venoms (Olamendi-Portugal et al., 2002; Shahbazzadeh et al., 2007; Fajloun et al., 2000) and further underscore the extensive molecular diversity of *A. mauritanicus* venom, particularly in neurotoxins targeting ion channels. This diversity is reflected not only in the polymorphism of the identified families but also in the variety of membrane receptors and ion channels they modulate. The identification of novel peptides, especially those targeting ion channels, highlights the unexplored biotechnological potential of this venom and opens new avenues for therapeutic development (Díaz-García and Varela, 2020).

Arachnid venoms, employed as tools for both defense and predation, serve to kill or immobilize prey for feeding or to neutralize competitors and potential predators. These venoms exhibit remarkable molecular diversity and complexity, with the expression of proteins and peptides governed by intricate gene regulation mechanisms that are still under investigation (Suranse et al., 2018; Marchi et al., 2022).

Scorpion venoms have been extensively studied, primarily due to their clinical effects on humans, which can sometimes lead to fatal outcomes (Chippaux and Goyffon, 2008). Paradoxically, deeper insights into the mechanisms of action of venom components have paved the way for biotechnological applications, with many research efforts focused on developing novel therapeutics based on the structure and function of these molecules (Rates et al., 2011). Despite its potential, *A. mauritanicus* venom remains an underexplored source of novel proteins that could contribute to biotechnological advancements.

The rapid expansion of identified scorpion venom compounds has revealed several promising drug candidates to address emerging global medical challenges (Hmed et al., 2013). Biologically active peptides from scorpion venoms are broadly classified into disulfide-bridged peptides (DBPs) (Lavergne et al., 2015) and non-disulfide-bridged peptides (NDBPs) (Almaaytah and Albalas, 2014; Zeng et al., 2005). Notably, DBPs represent the major components responsible for the neurotoxic symptoms observed in cases of scorpion envenomation (Mata et al., 2017).

Scorpion venom presents promising therapeutic potential for the treatment of infectious diseases. Several antiviral molecules, including neurotoxins and DBPs, have been identified from these venoms (Table 7) (Li et al., 2011). DBPs typically consist of approximately 30 amino acids, with three or four disulfide bridges arranged in a cysteine-stabilized motif (CS-), where a loop between two strands mimics the CDR2 loop of the CD4 receptor (Mata et al., 2017). DBPs can bind to the gp120 glycoprotein of HIV through molecular mimicry of CD4^+^ receptors on host cells. This interaction disrupts the gp120-CD4 binding, thereby preventing viral entry into host cells (Quinlan et al., 2014). Furthermore, scorpion potassium channel toxins, such as charybdotoxin (ChTx) and scyllatoxin, exhibit similar activity in blocking the gp120-CD4 interaction. Interestingly, mucroporin and its derivative mucroporin-M1, both with enhanced positive charge, interact directly with viral envelopes and have demonstrated antiviral effects against SARS-CoV and H5N1 viruses (Li et al., 2011).

Consistent with the antiviral potential of scorpion venoms, the non-disulfide-bridged peptide (NDBP) Ctry2459, isolated from the venom gland of *Chaerilus tryznai*, was shown to inhibit initial HCV infection in Huh7.5.1 cells by directly inactivating infectious viral particles (Mata et al., 2017). However, the 13-amino-acid peptide displayed limited bioavailability and was unable to suppress established infection. To overcome this limitation, histidine-rich analogs derived from the Ctry2459 scaffold—Ctry2459-H2 and Ctry2459-H3—were engineered to improve helicity, amphiphilicity, and endosomal escape. These modified peptides exhibited enhanced antiviral activity, reducing intracellular viral RNA by 40% and 70%, respectively, whereas the parental peptide mainly affected viral infectivity without significantly lowering intracellular viral levels (El Hidan et al., 2021). In comparison, crude venoms from *S. maurus palmatus* and *A. australis* have also demonstrated antiviral activity against HCV, with IC50 values of 6.3 ± 1.6 and 88.3 ± 5.8 μg/mL, respectively. Notably, the venom of *Scorpio maurus palmatus* reduces viral infectivity via a virucidal mechanism targeting the entry step, without affecting intracellular viral replication, and its activity is resistant to metalloprotease inhibition or heat treatment at 60 °C. In contrast, the Ctry2459-derived peptides represent a novel class of NDBPs capable not only of virucidal action but also of reducing intracellular viral RNA, highlighting their potential as more versatile antiviral agents compared with previously reported scorpion venom-derived compounds such as those from *A. australis* (El-Bitar et al., 2015; Mata et al., 2017).

The COVID-19 pandemic has emphasized the importance of vaccination in controlling the spread of SARS-CoV-2. However, the challenges posed by delays in vaccine production and distribution, along with the emergence of viral variants partially resistant to natural or vaccine-induced immune responses, highlight the need for additional preventive *mauritanicus* and therapeutic strategies. Combining vaccination with other measures such as diagnostics, protective protocols, and novel treatments remains essential. Developing new therapeutic agents, particularly those that inhibit viral entry, could offer significant benefits in reducing transmission and mitigating severe cases of infection. Recent research has turned to bioactive molecules from venomous animals, such as scorpions, to explore their potential as antiviral agents. Venom-derived molecules, known for their diverse pharmacological properties, have emerged as valuable candidates for therapeutic development.

This research focused on peptides isolated from Moroccan scorpion *A. mauritanicus*, to identify potential antiviral agents targeting SARS-CoV-2. The spike (S) protein of the virus, which plays a crucial role in its entry into host cells by binding to the ACE2 receptor (Lan et al., 2020). Our fraction’s antiviral potential was validated through ELISA analysis, uncovering five promising candidates with two demonstrating strong binding affinity (Figure 4). These fractions suggest a potential mechanism of viral entry inhibition, the peptides present in fractions F29 and F34 may directly bind to the receptor-binding domain (RBD) of the Spike protein, thereby preventing its interaction with the human ACE2 receptor. This specific inhibitio provides a plausible mechanistic hypothesis that nevertheless requires further functional validation.

The identification of 507 distinct molecular masses in *A. mauritanicus* venom, including 55 novel NaScTxs and ion channel-targeting peptides, significantly expands the known pharmacopeia of scorpion-derived bioactive compounds. These findings are particularly relevant given the urgent need for novel antiviral strategies, as exemplified by the COVID-19 pandemic. The discovery of fractions (F19–F29) with high-affinity binding to SARS-CoV-2 spike protein suggests a potential mechanism for viral entry inhibition, mirroring the gp120-CD4 blockade observed in HIV by other scorpion DBPs (Quinlan et al., 2014). This work provides the first evidence that *A. mauritanicus* peptides may interfere with ACE2-S protein interactions, offering a template for developing peptide-based antivirals against emerging coronaviruses. Furthermore, the heterogeneous neurotoxin profiles (e.g., α-toxins Amm3/5) highlight untapped opportunities for ion channel research, with potential applications in pain management and neurological disorders.

The study’s major strength lies in its multi-platform mass spectrometry approach, which enabled detection of low-abundance peptides (e.g., CaScTxs) through Q-Exactive LC/MS’s superior HCD sensitivity (Fedorova et al., 2013). However, key limitations must be acknowledged; *A. mauritanicus* venom procurement remains challenging due to the species’ endangered status in Morocco and low venom yields (∼0.5 mg per milking) (Oukkache et al., 2013), restricting large-scale studies. Limited fragmentation efficiency for long toxins during sequence coverage (e.g., 9% coverage for Lipolysis-activating peptide) underscores the need for hybrid techniques like ETD-MS/MS in future work. While ELISA confirmed S-protein binding, it is important to note that the RBD–ACE2 binding assay has been validated in previous studies to predict antiviral efficacy. For instance, it was demonstrated that compounds such as zafirlukast, identified by their ability to inhibit S1RBD–ACE2 binding, effectively blocked the entry of SARS-CoV-2 pseudovirus into cells (Zhang et al., 2022). These findings support the idea that inhibition of the ACE2–RBD interaction is a critical step that can lead to antiviral effects. Nevertheless, further assays using viral or pseudoviral infection models are recommended to fully confirm the antiviral potential of the identified venom fractions. *In vivo* efficacy and toxicity profiles of lead peptides (e.g., F19–F22) also remain to be characterized.

Our research highlights the safety profiles *in vivo* of fractions 29 and 34 as promising antiviral candidates. The absence of neurotoxic symptoms and lethality suggests that these fractions can effectively inhibit viral entry without inducing adverse neurological effects, a critical consideration for therapeutic development (Table 9). Nevertheless, further *in vivo* studies are required to evaluate their systemic efficacy, pharmacokinetics, and long-term safety in relevant infection models. Such investigations will be essential to fully characterize their therapeutic potential and ensure translational relevance.

This study establishes *A. mauritanicus* venom as a rich source of both neurotoxins and antiviral candidates, but translational success will require interdisciplinary collaboration between toxinology, virology, and drug delivery fields. Rational design of truncated analogs (e.g., mucroporin-M1 derivatives (Mata et al., 2017) could enhance ACE2-binding affinity while reducing neurotoxicity. Evaluating fractions against viral variants (e.g., Omicron sublineages) and other enveloped viruses (e.g., influenza) given conserved targeting of host receptors. Nanocarrier encapsulation could address peptide stability issues, as demonstrated for chlorotoxin glioma therapies (Costa et al., 2013). Recombinant expression of high-priority toxins (e.g., α-KTx 9.1 homologs) in *E. coli* or yeast to circumvent venom supply constraints (King and Hardy, 2013). This interdisciplinary enrichment of our results and the development of this research is encouraged.

## 5 Conclusion and perspectives

This work not only demonstrates the molecular diversity and therapeutic potential of scorpion venoms but also highlights the importance of innovative approaches in addressing emerging viral threats. As global efforts continue to develop effective COVID-19 treatments, venom-derived peptides could play a pivotal role in complementing existing strategies and mitigating future outbreaks.

This study focused on mapping the proteome of *A. mauritanicus* venom, the species most frequently associated with severe envenomation in Morocco. The aim was to investigate the molecular diversity and toxic components of the venom to develop an effective antivenom, while also identifying novel molecules with potential for therapeutic or biotechnological applications. Analysis of the venom after fractionation revealed its complex nature, comprising a broad range of components, with neurotoxins primarily targeting sodium (NaScTxs) and potassium channels (KscTxs). It was found that the venom is particularly rich in NaScTxs, which act on mammalian sodium channels, explaining its role in the most fatal scorpion envenomations reported in the region.

Moreover, the venom also contains neurotoxins that affect chloride channels (ClScTxs), further demonstrating the vast diversity of its toxic arsenal. This diversity is reflected both in the molecular variability within toxin families and in the variety of targeted receptors and ion channels, some of which may offer promising avenues for pharmaceutical development. The findings presented in this paper not only shed light on the molecular diversity of *A. mauritanicus* venom but also contribute to the growing field of venom-based therapeutics. The identification of peptides with inhibitory effects on SARS-CoV-2 provides a foundation for developing new antiviral drugs, offering a complementary strategy to vaccination and other treatments. However, claims regarding potential therapeutic or industrial applications must be interpreted cautiously. The antiviral activity reported here is based on *in vitro* ELISA assays targeting the Spike–ACE2 interaction, and further validation using pseudoviral or authentic SARS-CoV-2 infection models is necessary to confirm efficacy, elucidate mechanisms of action, and assess *in vivo* safety. These findings therefore highlight promising candidates for future studies rather than immediate therapeutic use, emphasizing the need for additional research to explore their full potential.

## Data Availability

Raw data are available via ProteomeXchange (PRIDE) with identifier PXD069182. Available at: https://proteomecentral.proteomexchange.org/cgi/GetDataset?ID=PXD069182.
